# Patient-derived enteroids provide a platform for the development of therapeutic approaches in microvillus inclusion disease

**DOI:** 10.1172/JCI169234

**Published:** 2023-10-16

**Authors:** Meri Kalashyan, Krishnan Raghunathan, Haley Oller, Marie-Theres Bayer, Lissette Jimenez, Joseph T. Roland, Elena Kolobova, Susan J. Hagen, Jeffrey D. Goldsmith, Mitchell D. Shub, James R. Goldenring, Izumi Kaji, Jay R. Thiagarajah

**Affiliations:** 1 Division of Gastroenterology, Hepatology and Nutrition, Boston Children’s Hospital, Harvard Medical School, Boston, Massachusetts, USA.; 2 Congenital Enteropathy Program, Boston Children’s Hospital, Boston, Massachusetts, USA.; 3 PediCODE Consortium, as detailed in Supplemental Acknowledgments.; 4 Section of Surgical Sciences and; 5 Epithelial Biology Center, Vanderbilt University Medical Center, Nashville, Tennessee, USA.; 6 Department of Surgery, Division of Surgical Sciences, Beth Israel Deaconess Medical Center and Harvard Medical School, Boston, Massachusetts, USA.; 7 Department of Pathology, Boston Children’s Hospital, Harvard Medical School, Boston, Massachusetts, USA.; 8 Department of Child Health, University of Arizona College of Medicine–Phoenix, and Division of Gastroenterology, Phoenix Children’s Hospital, Phoenix, Arizona, USA.; 9 Nashville VA Medical Center, Nashville, Tennessee, USA.; 10 Harvard Digestive Disease Center, Boston, Massachusetts, USA.

**Keywords:** Gastroenterology, Drug therapy, Epithelial transport of ions and water, Genetic diseases

## Abstract

Microvillus inclusion disease (MVID), caused by loss-of-function mutations in the motor protein myosin Vb (MYO5B), is a severe infantile disease characterized by diarrhea, malabsorption, and acid/base instability, requiring intensive parenteral support for nutritional and fluid management. Human patient–derived enteroids represent a model for investigation of monogenic epithelial disorders but are a rare resource from MVID patients. We developed human enteroids with different loss-of function MYO5B variants and showed that they recapitulated the structural changes found in native MVID enterocytes. Multiplex immunofluorescence imaging of patient duodenal tissues revealed patient-specific changes in localization of brush border transporters. Functional analysis of electrolyte transport revealed profound loss of Na^+^/H^+^ exchange (NHE) activity in MVID patient enteroids with near-normal chloride secretion. The chloride channel–blocking antidiarrheal drug crofelemer dose-dependently inhibited agonist-mediated fluid secretion. MVID enteroids exhibited altered differentiation and maturation versus healthy enteroids. γ-Secretase inhibition with DAPT recovered apical brush border structure and functional Na^+^/H^+^ exchange activity in MVID enteroids. Transcriptomic analysis revealed potential pathways involved in the rescue of MVID cells including serum/glucocorticoid-regulated kinase 2 (SGK2) and NHE regulatory factor 3 (NHERF3). These results demonstrate the utility of patient-derived enteroids for developing therapeutic approaches to MVID.

## Graphical abstract

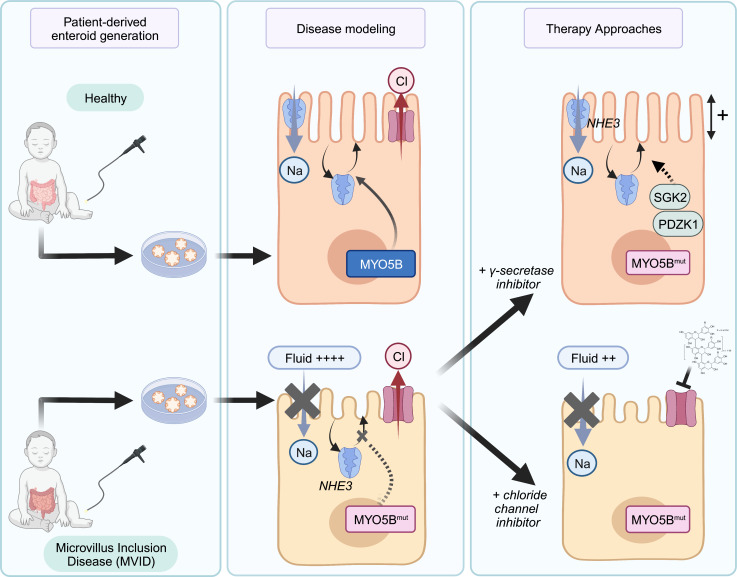

## Introduction

Microvillus inclusion disease (MVID) is a rare congenital diarrhea and enteropathy (CoDE) caused by loss-of-function mutations in the motor protein myosin Vb (MYO5B) (1–5). In general, patients with severe loss of MYO5B function have profound diarrhea, intestinal fluid loss, and nutrient malabsorption requiring parenteral nutrition and supplemental fluids to maintain growth and fluid status (4, 6). Management of MVID is difficult, with several clinical problems, including acidosis, dehydration, central line infections, and micronutrient deficiency. Previously MVID was often lethal in infancy, but advances in parenteral nutrition have extended life expectancy beyond early childhood (7, 8). Nevertheless, management of MVID is currently primarily supportive and sometimes proceeds to intestinal transplant, with no effective symptomatic or disease-modifying therapies.

MYO5B function is critical for normal polarized vesicular trafficking in intestinal epithelial cells and particularly important for protein recycling at the intestinal apical membrane (1, 9–14). Loss of MYO5B function leads to defective microvillus formation and a structurally abnormal apical brush border (1, 11). MYO5B activity is thought to be critical for normal localization of intestinal transport proteins involved in nutrient and fluid absorption and secretion (15–17). Previous studies in *MYO5B*-knockout animal models and intestinal cancer cell lines have shown profound loss or mislocalization of key transporters such as the intestinal sodium-dependent glucose cotransporter (SGLT1) and sodium/hydrogen exchanger 3 (NHE3) from the apical membrane, with intact cystic fibrosis transmembrane conductance regulator (CFTR) localization (16). Several recent studies examining human MVID intestinal tissues have confirmed these protein changes, but the functional balance of fluid absorption versus secretion in MVID patient cells remains untested (18). Although animal models and transformed cell studies have been important in our current understanding of MVID pathophysiology, the ability to extrapolate and understand MYO5B-related changes in primary human cells and tissues remains a priority.

Human intestinal stem cell–derived enteroids obtained from endoscopic mucosal biopsy tissue represent a potentially powerful model for understanding protein function and phenotype-genotype correlations and for therapy development in monogenic epithelial disorders such as MVID (19, 20). Previous studies in genetic disorders affecting the intestine such as cystic fibrosis (21) and TTC7A deficiency (22) have highlighted the use of phenotypic and functional assessments for developing and validating therapies and providing patient-specific preclinical data. A major barrier to enteroid generation for MVID, however, is that patients rarely undergo endoscopy after initial diagnosis. This along with a need for access to a tertiary medical center with the research resources and logistics required for enteroid generation has led to a lack of availability of human primary enteroids for the study of MVID.

Differentiation and maturation of intestinal epithelial cells from intestinal stem cell–derived progenitor populations into specialized cell types including secretory/sensory cells such as goblet, enteroendocrine, and tuft cells as well as mature absorptive enterocytes are dependent on the balance of secreted growth factors that activate Wnt, Notch, and TGF-β signaling pathways (23). Previous studies in MVID mouse models have indicated that loss of MYO5B function may lead to altered differentiation and maturation of intestinal cells and that altering intestinal Wnt/Notch balance may be a potential therapeutic strategy (24).

Here, we report the generation and functional investigation of human small intestinal enteroids derived from 2 patients with MVID due to loss-of-function *MYO5B* mutations. We show that MVID patient–derived enteroids recapitulate the native epithelial tissue phenotype both structurally and functionally, with loss of microvilli, loss of sodium-dependent electrolyte absorption, and intact chloride secretion and fluid secretion. We leverage these readouts of transport function to assess the potential utility of the antisecretory antidiarrheal drug crofelemer for symptomatic therapy for MVID. We further show that loss of MYO5B function leads to defective epithelial differentiation and maturation and that γ-secretase inhibition can structurally and functionally rescue MVID enterocytes. Lastly, we carried out transcriptomic analysis of enteroids following γ-secretase inhibition, revealing pathways and targets for potential disease-modifying therapies for MVID.

## Results

### Enteroids from MVID patients have a defective brush border and altered differentiation.

Duodenal endoscopic biopsies were obtained from 2 patients with severe intestinal manifestations of MVID (see Supplemental Methods; supplemental material available online with this article; https://doi.org/10.1172/JCI169234DS1) along with age-matched heathy controls and enteroids generated as previously described (25, 26). Patient 1 was confirmed to have compound heterozygous variants in the *MYO5B* gene: c.2111delA (p.Phe704Serfs66*), a frameshift mutation, and c.1576G>A (p.Q526*), a paternally inherited novel nonsense mutation that leads to early truncation. These early truncation mutations lead to a functional MYO5B deletion, so the mutations in this patient are referred to as MYO5B KO (Supplemental Figure 1A). Patient 2 had a confirmed homozygous missense mutation in *MYO5B*: c.1979C>T (p.P660L), a known missense mutation that leads to loss of motor protein function (referred to as P660L in the text) (10).

**Figure 1:**
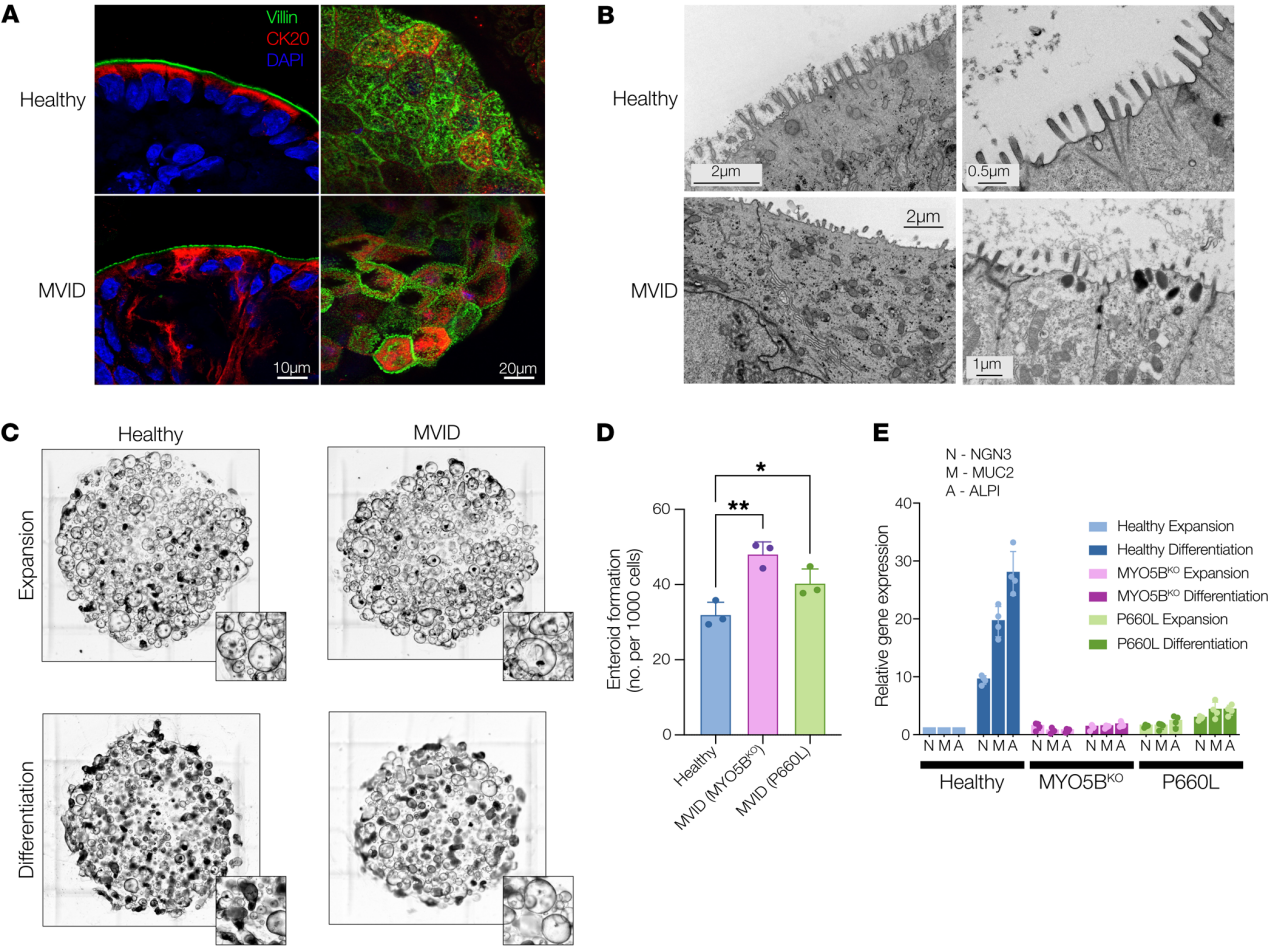
MVID enteroids recapitulate native epithelial disease changes. (**A**) Representative confocal images of villin (green), cytokeratin 20 (CK20, red), and nuclei (DAPI) of healthy and MVID (MYO5B^KO^) enteroids in cross section (left; scale bar: 10 μm) or en face (right; scale bar: 20 μm). Representative of 3 separate samples. (**B**) Representative electron micrographs of healthy and MVID enteroids (MYO5B^KO^). Representative of 2 separate samples. Scale bars: left, 2 μm; top right, 0.5 μm; bottom right, 1 μm. (**C**) Bright-field images of enteroid cultures grown in expansion versus differentiation medium. Inset images highlight changes in cultures with increased spheroid (stem-like) morphology in MVID differentiated cultures versus healthy. Representative of 6 separate experiments. (**D**) Enteroid formation assay showing new enteroid formation (at 4 days after plating) following enzymatic dissociation and replating. Data are shown as means ± SEM; *n* = 3 experiments; *P < 0.05, **P < 0.01; 2-way ANOVA with Tukey’s post hoc testing. (**E**) Relative gene expression (normalized to healthy expansion) for neurogenin 3 (NGN3), mucin 2 (MUC2), and alkaline phosphatase (ALPI) in healthy and MVID enteroids following switching to enteroid differentiation medium. Data are shown as means ± SD; *n* = 4 experiments; 2-way ANOVA with Tukey’s post hoc testing.

Confocal and electron microscopy of enteroids (Figure 1, A and B, and Supplemental Figure 2) indicated loss of apical microvilli and reduced epithelial cell height in MVID cells as compared with a healthy control. MVID enteroids (MYO5B KO and P660L) were grown in standard high-Wnt-containing enteroid expansion medium (WERN) and passaged similarly to healthy controls, but also showed an increased ability to be expanded and form new enteroids when passaged (Figure 1, C and D). When switched to low-Wnt differentiation medium, healthy control enteroids showed robust differentiation and maturation (Figure 1, C and E) with increased expression of markers of mature secretory and absorptive epithelial cells (Muc2, Ngn3, Alpi). In contrast, both MVID enteroid lines had markedly attenuated epithelial marker transcription, suggesting that loss of MYO5B function is associated with altered epithelial cell maturation. The appearance of pathognomonic intracellular inclusions was generally rare in the human MVID enteroids and was only noted occasionally in differentiated (low-Wnt medium) cultures (Supplemental Video 1).

### MVID mutations result in altered secretory cell populations.

**Figure 2:**
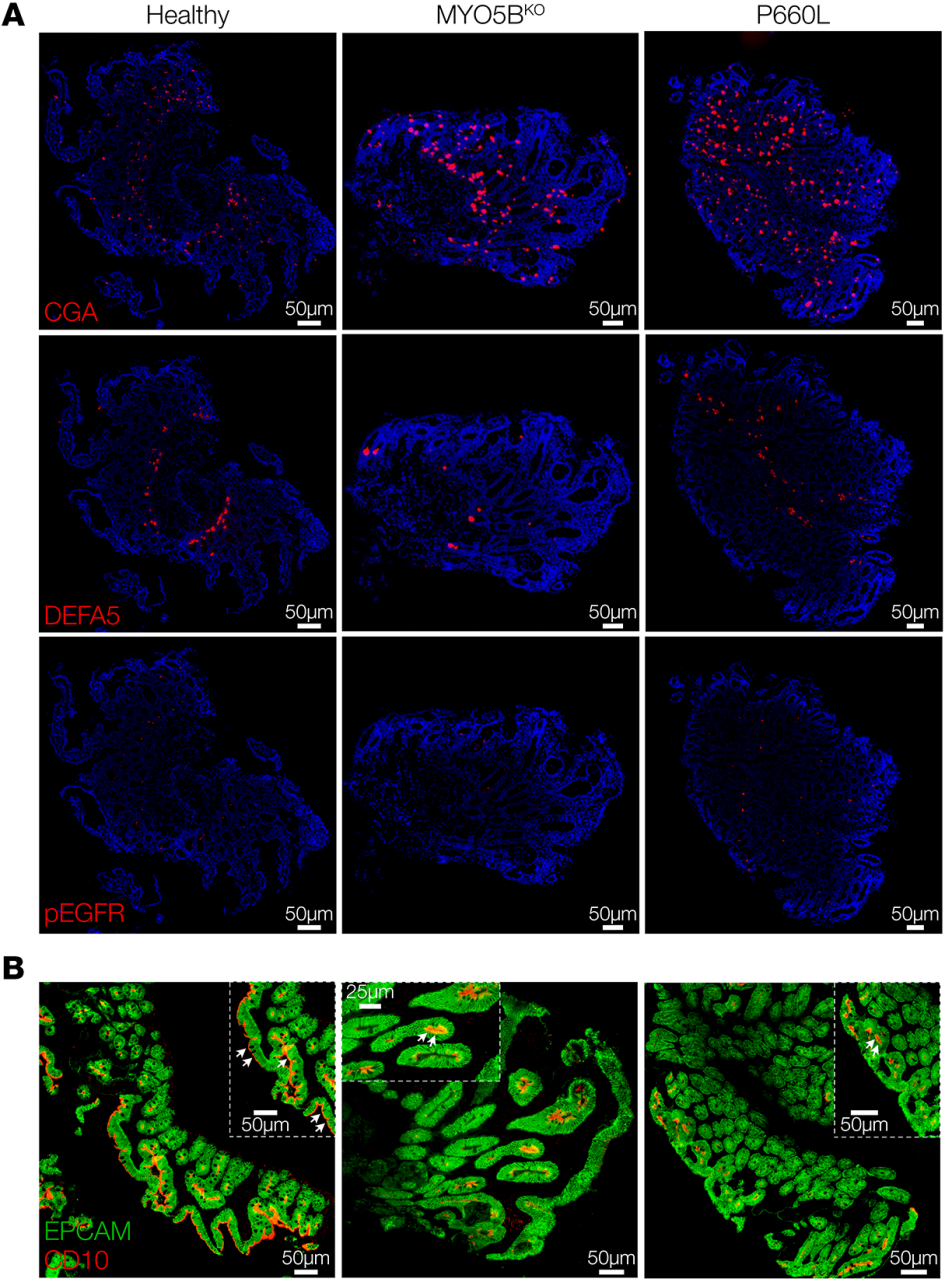
Altered secretory cell populations in MVID patient tissues. (**A**) Immunofluorescence images of human duodenal biopsy sections stained for chromogranin A (CGA), defensin α5 (DEFA5), and phospho–epidermal growth factor receptor (p-EGFR). Representative of 3 separate sections per tissue. Scale bars: 50 μm. (**B**) Images of CD10 and epithelial cell adhesion molecule (EPCAM) showing reduced linear CD10 staining in MVID tissues. Representative of 3 separate sections per tissue. Scale bars: 50 μm, except middle inset, 25 μm.

To further assess cell populations and protein localization in MVID, we conducted multiplex immunofluorescence (MxIF) staining (27) and analysis of MVID patient duodenum compared with healthy control tissue. MVID tissues from both patients showed reduced numbers of phospho-EGFR^+^ tuft cells and defensin α5^+^ Paneth cells, increased chromogranin A^+^ enteroendocrine cells, and similar numbers of goblet cells (Figure 2A, Supplemental Figure 1B, and Supplemental Figure 3). Apical enterocyte alterations in MVID can be patchy with areas of intact but discontinuous brush border. MVID enteroids (Figure 1A) also exhibited a patchy loss of apical brush border villin staining. To provide quantification of brush border continuity in tissue sections, we analyzed staining for CD10, an aminopeptidase and a well-known marker of the brush border in mature enterocytes, by skeletonizing positive signal and measuring line distances (mean Feret distance). Using this metric, we found as expected that MVID tissues from both patients showed reduced brush border continuity versus controls (Figure 2B and Supplemental Figure 1).

### MxIF analysis reveals variant-specific alterations in enterocyte transport protein localization in MVID small intestine.

**Figure 3:**
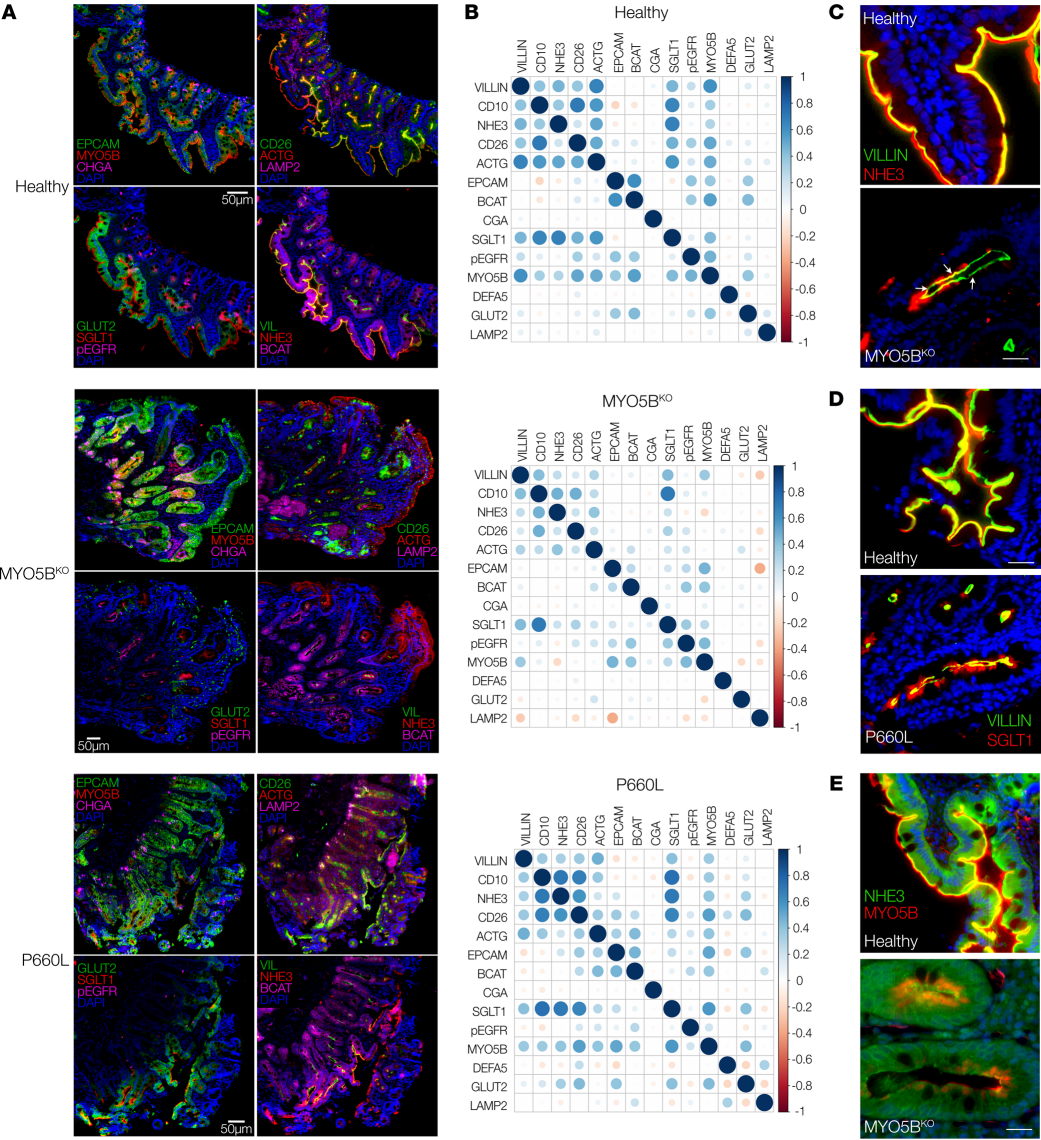
Multiplex immunofluorescence highlights protein localization changes in patient tissues. (**A**) Multiplex immunofluorescence panels representing 12 antigens on duodenal biopsy tissues. Scale bars: 50 μm. The Healthy and MYO5B^KO^ panels include data from the same sample as in Figure 2A, MYO5B^KO^, rows 2 and 3, and Figure 2B, Healthy and MYO5B^KO^. (**B**) Cross-correlation matrices for paired antigens (Pearson’s coefficient) with dot color indicating direction of correlation and dot size indicating extent of correlation. (**C**–**E**) Individual pairwise staining for Na^+^/H^+^ exchanger 3 (NHE3) and villin (**C**), sodium-glucose cotransporter 1 (SGLT1) and villin (**D**), and myosin Vb (MYO5B) and NHE3 (**E**). Scale bars: 10 μm.

A major advantage of MxIF staining is the ability to assess multiple antigens on the same tissue section, allowing cellular protein-protein localization within the resolution of the imaging optics. We applied a targeted panel of antibodies that included epithelial cell type markers, structural markers, and important small intestinal transport proteins (Figure 3A). We conducted a cross-correlation analysis to compare localization of the panel proteins from the 2 MVID patient tissues against a healthy control (Figure 3B). The overall pattern of correlations between specific pairs of antigens was more similar to the healthy control for the P660L mutation intestine versus the MYO5B KO. Correlations of membrane-associated transporters with mature enterocyte markers of apical and basolateral membranes were reduced in both MVID patients (NHE3-villin and GLUT2–β-catenin; Figure 3C and Supplemental Figure 4). Although SGLT1 showed similar correlations with the apical membrane in healthy and MVID tissues, closer inspection of villus tip enterocytes revealed reduced apical staining and increased internalization in MVID tissues (SGLT1-villin; Figure 3D). In contrast, NHE3 showed both reduced abundance and reduced membrane localization (NHE3-villin; Figure 3C) and markedly altered and mutation-dependent association and localization with MYO5B (Figure 3E). These MxIF tissue studies corroborate previous findings of reduced apical transport proteins and alterations in cellular localization in MVID and allow future analysis of genotype-specific changes.

### Epithelial sodium/hydrogen exchange is severely impaired but chloride secretion is intact in human MVID.

**Figure 4:**
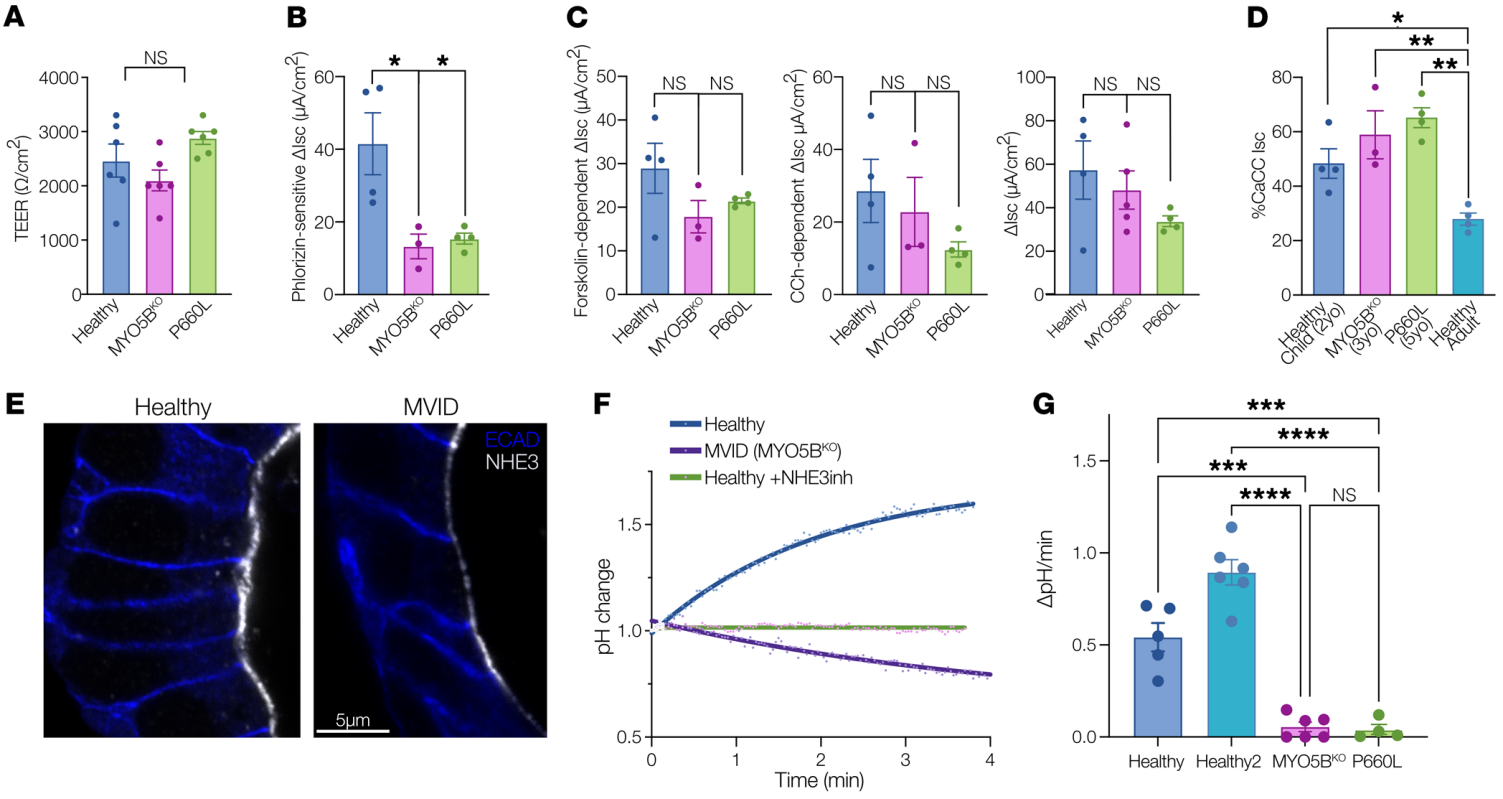
Loss of sodium absorption and normal chloride secretion in MVID patient enteroids. (**A**) Transepithelial electrical resistance (TEER) 10–14 days after plating on Transwell inserts. Data are shown as means ± SEM; *n* = 6 monolayers. (**B**) Glucose-stimulated (20 mM), phlorizin-inhibitable short-circuit current (*I*_sc_) in healthy and MVID monolayers. Data are shown as means ± SEM; *n* = 3–4 monolayers; *P < 0.05. (**C**) Left: *I*_sc_ stimulated by 10 μM forskolin. Data are shown as means ± SEM; *n* = 3–4 monolayers. Middle: *I*_sc_ stimulated by 50 μM carbachol (CCh). Data are shown as means ± SEM; *n* = 3–4 monolayers. Right: *I*_sc_ stimulated by combined forskolin and CCh. Data are shown as means ± SEM; *n* = 3–4 monolayers. (**D**) Percentage CCh- (50 μM) and forskolin- (10 μM) stimulated *I*_sc_ inhibited by CaCC inhibitor A01 (50 μM) in healthy control cells grown from a young donor (2 years old), an adult donor (20 years old), and MVID patients (3 and 5 years old). Data are shown as means ± SEM; *n* = 3–4 monolayers; *P < 0.05, **P < 0.01. (**E**) Super-resolution images (stimulated emission depletion [STED]) of NHE3 localization and abundance in healthy and MVID patient enteroids. Scale bar: 5 μm. (**F**) Example curves showing change in pH calculated from analysis of intensity changes of SNARF-5F fluorescence in healthy and MVID cells and healthy cells after pretreatment with the NHE3 inhibitor (10 μM). (**G**) Summary graph showing NHE3-dependent pH changes in healthy and MVID patient cells. Data are shown as means ± SEM; *n* = 4–6 experiments; ***P < 0.001, ****P < 0.0001; 2-way ANOVA with Tukey’s post hoc testing.

To functionally assess electrolyte absorption and secretion in human patient MVID epithelial cells, we conducted a series of electrophysiological and fluorometric cellular transport experiments. Patient-derived intestinal cells were cultured as differentiated monolayers on Transwell inserts, and transepithelial short-circuit current (*I*_sc_) was measured using a custom-built multi-well Ussing chamber (Supplemental Methods). MVID cells had a transepithelial electrical resistance similar to that of healthy controls at the time of measurement (Figure 4A). Phlorizin-sensitive glucose-stimulated *I*_sc_, reflecting SGLT1 transport, was reduced in MVID cells by approximately 60% in comparison with controls (Figure 4B). In contrast, chloride secretion induced by both forskolin (cAMP) and carbachol (Ca^2+^) was similar between MVID cells and controls (Figure 4C). Chloride secretion stimulated by the muscarinic agonist carbachol was notably high in both control and MVID primary duodenal cells. Carbachol-stimulated chloride currents in intestinal cell lines (e.g., T84 cells) are known to be predominantly mediated via CFTR. To further assess this, we measured agonist-stimulated currents with CFTR (CFTR_inh_-172) (28) and calcium-activated chloride channel (CaCC; CaCC_inh_-A01) (29) inhibition in young healthy and MVID duodenal cells versus cells derived from an adult grown in the same conditions (Figure 4D). Interestingly, the contribution of non-CFTR, likely CaCC transport was significantly higher in early-childhood duodenal cells than in adult duodenal cells, suggesting important age-dependent differences in transport function.

Similarly to MxIF tissue staining, confocal imaging of enteroids showed markedly reduced localization of NHE3 at the apical membrane of MVID cells (Figure 4E). To functionally assess electroneutral Na^+^/H^+^ exchange, enteroids were grown on glass coverslips for fluorescence imaging. Cells were loaded with the intracellular pH-sensitive fluorophore SNARF-5F and placed in an imaging chamber. Using a previously optimized Na^+^-free alkalinization protocol, the kinetics of intracellular pH changes as a measure of NHE3 activity was monitored in patient-derived duodenal cells (30). As shown in Figure 4F, healthy control cells exhibited a robust change in pH that was blocked by pretreatment with an NHE3 inhibitor (S3226). MVID cells from both patients showed reduced and, in some experiments, complete loss of NHE3 activity (Figure 4G).

### An approved natural-compound antidiarrheal can inhibit chloride and fluid secretion in MVID epithelium.

**Figure 5:**
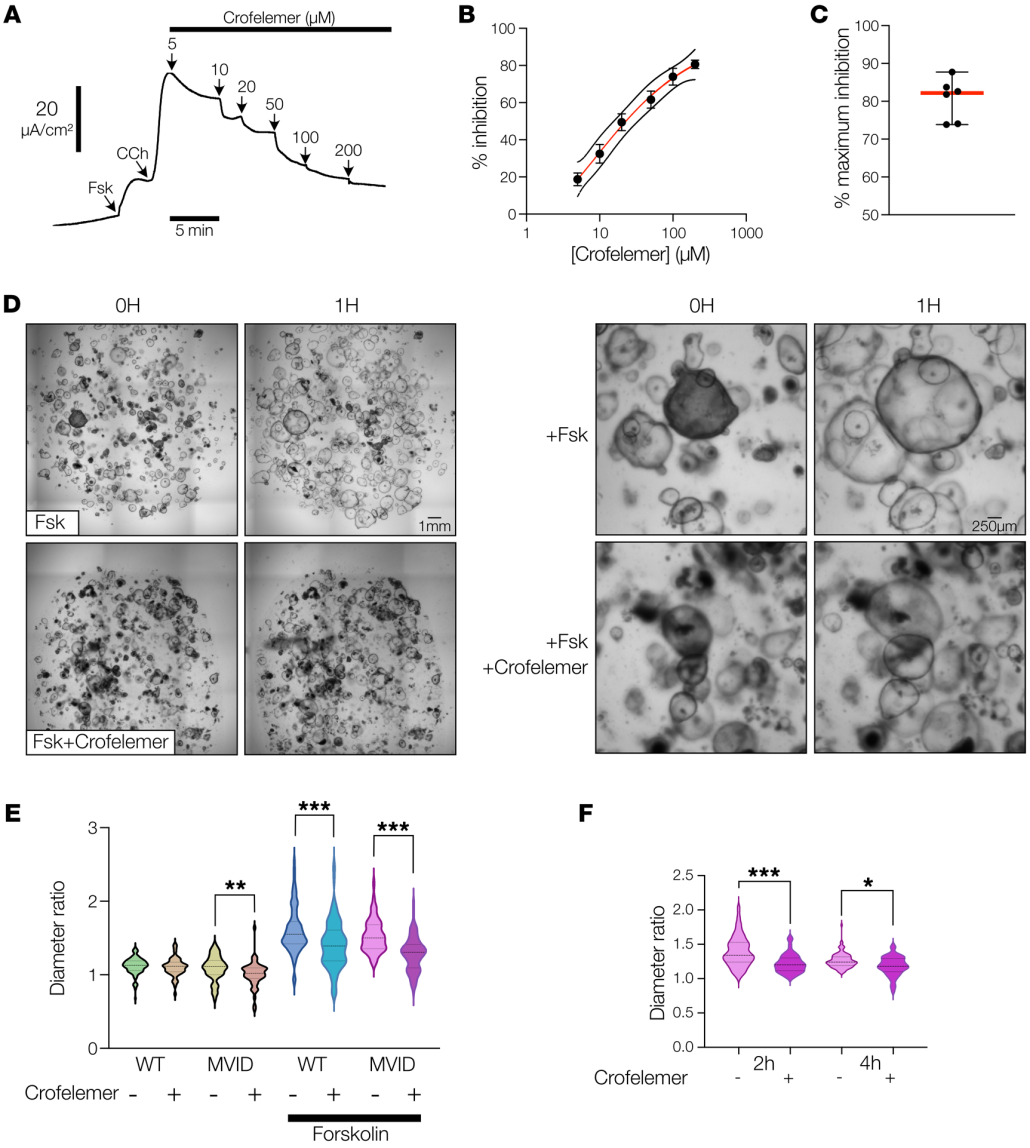
Crofelemer inhibits chloride and fluid secretion in MVID patient enteroids. (**A**) Representative curve showing dose-dependent inhibition of forskolin- (10 μM) and carbachol-stimulated (50 μM) *I*_sc_ by crofelemer. (**B**) Dose-response curve for crofelemer-induced inhibition of forskolin- and carbachol-stimulated *I*_sc_ in MVID patient enteroids (MYO5B KO). Data are shown as means ± SEM; *n* = 6 monolayers. (**C**) Maximal percentage inhibition of agonist-stimulated current by crofelemer. Data are shown as means ± SEM; *n* = 6 monolayers. (**D**) Example bright-field images before and after forskolin (2 μM) ± crofelemer (200 μM) in MVID patient enteroids. Right panels show higher magnification of enteroid swelling. Representative of 4 separate experiments. Scale bars: left panels, 1 mm; right panels, 250 μm. (**E**) Violin plot showing increase in enteroid size (diameter ratio) in healthy and MVID enteroids at 1 hour ± forskolin and ± crofelemer (200 μM). Dotted lines, median ± interquartile range; >300 enteroids from 4 experiments. (**F**) Diameter ratio in MVID enteroids at 2 and 4 hours after stimulation ± crofelemer (200 μM). Dotted lines, median and interquartile range; *n* = 3 experiments; *P < 0.05, **P < 0.01, ***P < 0.001; 2-way ANOVA with Tukey’s post hoc testing.

MVID patients can have very large daily fluid loss (2–5 L/d), with consequent substantial requirement for fluid replacement and a tenuous acid/base status. Our functional assessment of MVID cell transport showed severe loss of Na^+^-mediated fluid absorption in the setting of potentially intact chloride-mediated fluid secretion, suggesting that blocking chloride transport may theoretically improve fluid balance in MVID. To further assess this, we obtained the FDA-approved antidiarrheal compound crofelemer to test in MVID patient cells. Crofelemer is a proanthocyanidin, natural antidiarrheal reagent that inhibits both CFTR- and CaCC-mediated chloride secretion (31). Analysis of combined forskolin- and carbachol-stimulated *I*_sc_ showed a robust and dose-dependent inhibition with an IC_50_ of approximately 30 μM (Figure 5, A and B), similar to previous reports, but with an increased maximal inhibition compared with previous data in colonic cell lines (~80% vs. 50%) (Figure 5C) (31). To measure fluid secretion, we used a 3-dimensional enteroid swelling assay (21, 22) following stimulation of enteroid fluid secretion with forskolin and tested the efficacy of crofelemer (Figure 5D). Corroborating our chloride current data, both healthy and MVID enteroids exhibited a robust induction of fluid secretion, which was inhibited by approximately 40% and 50%, respectively, by administration of crofelemer (100 μM) with continued inhibition up to 4 hours after stimulation (Figure 5, E and F).

### γ-Secretase inhibition can structurally and functionally correct MVID epithelial defects.

**Figure 6:**
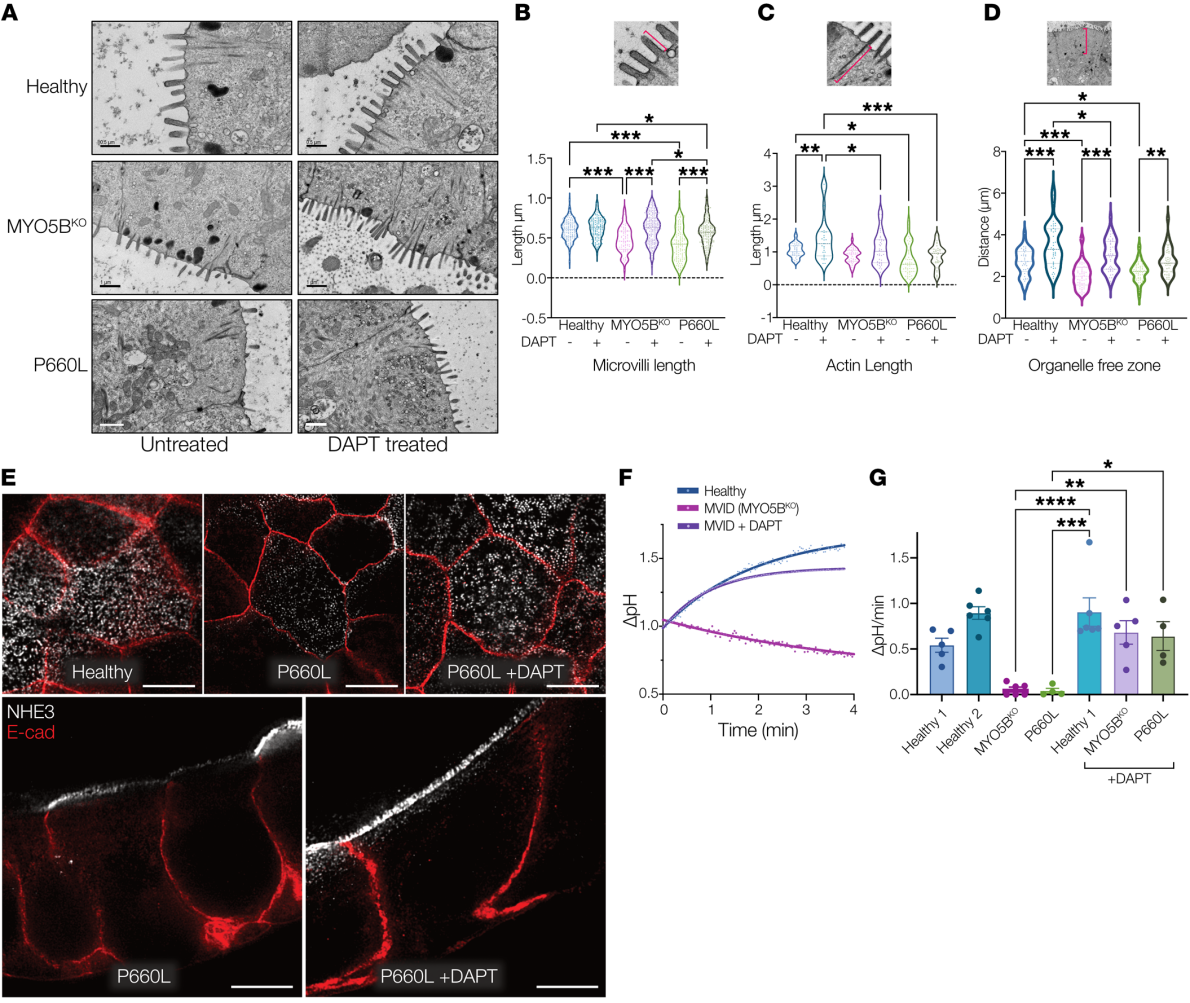
γ-Secretase inhibition rescues MVID patient enteroid differentiation. (**A**) Electron micrographs of healthy and MVID enteroids with and without DAPT treatment (10 μM). Scale bars: top panels, 0.5 μm; middle and bottom panels, 1 μm. (**B**) Analysis of electron microscopic images of microvillus length. (**C**) Subapical actin bundle length. (**D**) Distance of apical organelle free zone. Inset images above show example measured parameter. Graphs show measurements from at least 10 electron microscopic images. (**E**) Super-resolution confocal images (STED), en face (top) and cross section (bottom), of NHE3 localization and abundance in healthy and MVID patient enteroids following treatment with DAPT (10 μM). Scale bars: 5 μm. (**F**) Representative curves showing change in intracellular pH in healthy cells and MVID cells with and without DAPT. (**G**) Summary graph showing NHE3-dependent pH changes in healthy and MVID patient cells with and without DAPT. Data are shown as means ± SEM; *n* = 4–6 experiments; *P < 0.05, **P < 0.01, ***P < 0.001, ****P < 0.0001; 2-way ANOVA with Tukey’s post hoc testing.

Although attenuation of fluid losses would be of benefit for MVID patients, restoration of normal epithelial function including fluid absorption following MYO5B loss of function would have major implications for disease management. Previous studies in mouse models (16, 24) as well as our finding of impaired differentiation in patient enteroids indicated that alteration of Wnt/Notch signaling may allow functional epithelial recovery. Treatment of healthy enteroids with the γ-secretase inhibitor DAPT induced robust differentiation and polarization of cells (Figure 6A and Supplemental Figures 2 and 4). DAPT-treated MVID enteroids remarkably showed recovery of microvillus structure, with increased microvillus length (Figure 6B) and length of the actin core (Figure 6C). Overall cell polarization of MVID cells as measured by the organelle-free zone at the apical aspect of columnar epithelial cells was also increased following γ-secretase inhibition (Figure 6D). In addition to structural recovery, NHE3 abundance at the brush border was increased (Figure 6E), and there was significant recovery of NHE3 function as measured by direct Na^+^/H^+^ exchange activity of MVID cells from both patients (Figure 6, F and G).

### Transcriptomic analysis of γ-secretase inhibition in MVID enteroids reveals a target pathway for MVID therapy.

**Figure 7:**
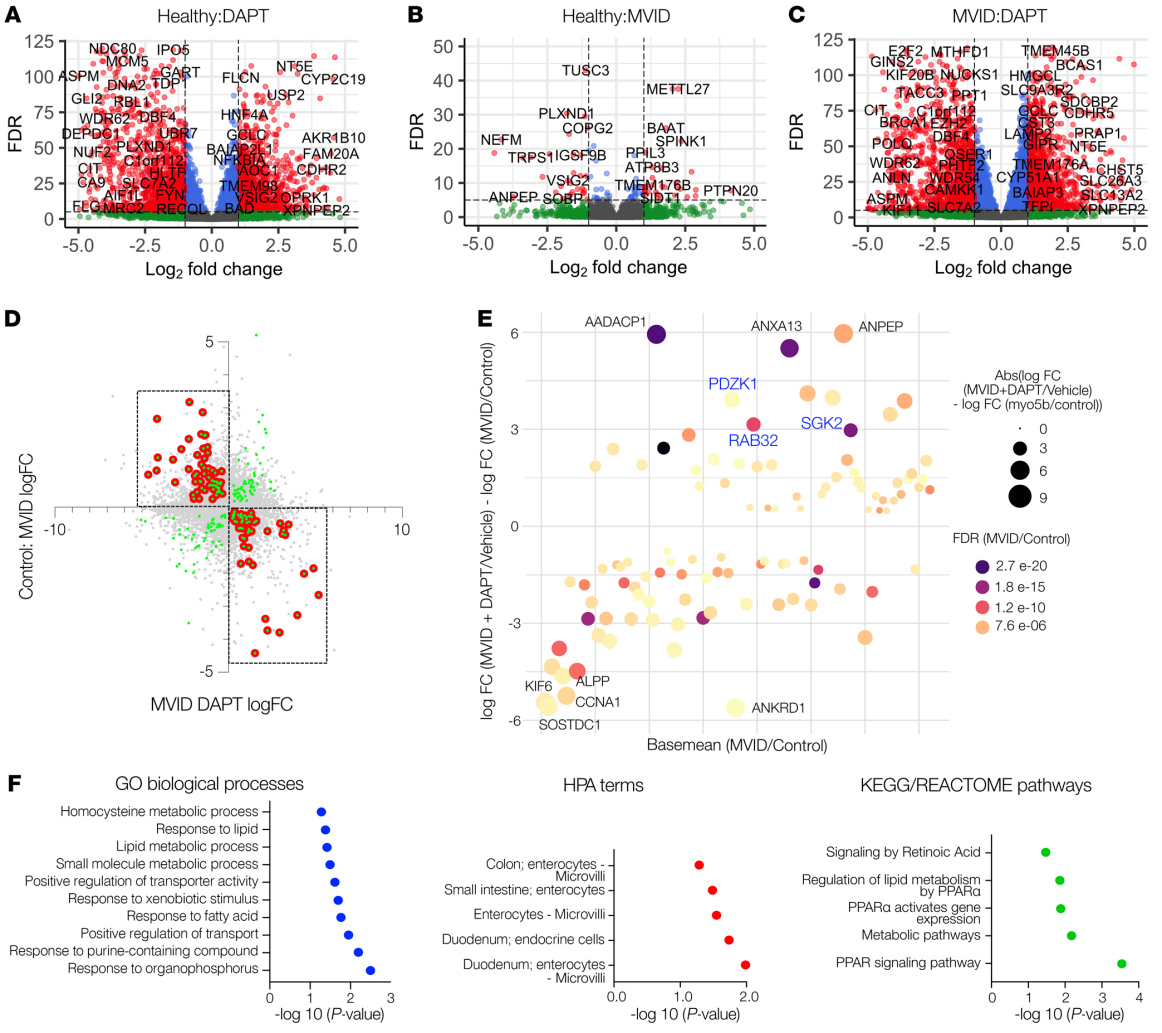
Genome-wide transcriptomic analysis reveals potential targets for rescue of MVID enteroids. (**A**) Volcano plot showing log_2_ fold change (FC) and false discovery rate (FDR) showing genes with significantly up- and downregulated expression (red) in healthy enteroids (*n* = 3) following DAPT treatment (10 μM). (**B**) Volcano plot showing genes with significantly up- and downregulated expression (red) between healthy enteroids and MVID enteroids (MYO5B KO) (*n* = 3). (**C**) Volcano plot showing genes with significantly up- and downregulated expression (red) in MVID enteroids (MYO5B KO) following DAPT treatment (10 μM). (**D**) Plot of genes with significantly altered expression (green dots) between MVID and healthy against MVID + DAPT. Red dots indicate genes changing in opposite directions following DAPT treatment (filtered genes). (**E**) Dot plot of filtered genes by change in expression and base mean expression, with dot size indicating fold change and color indicating FDR. Highlighted genes are based on previous functional data indicating a plausible biological role. (**F**) Pathway analysis showing most significant Gene Ontology (GO) terms, Human Protein Atlas (HPA) terms, and Kyoto Encyclopedia of Genes and Genomes (KEGG) pathways.

**Figure 8:**
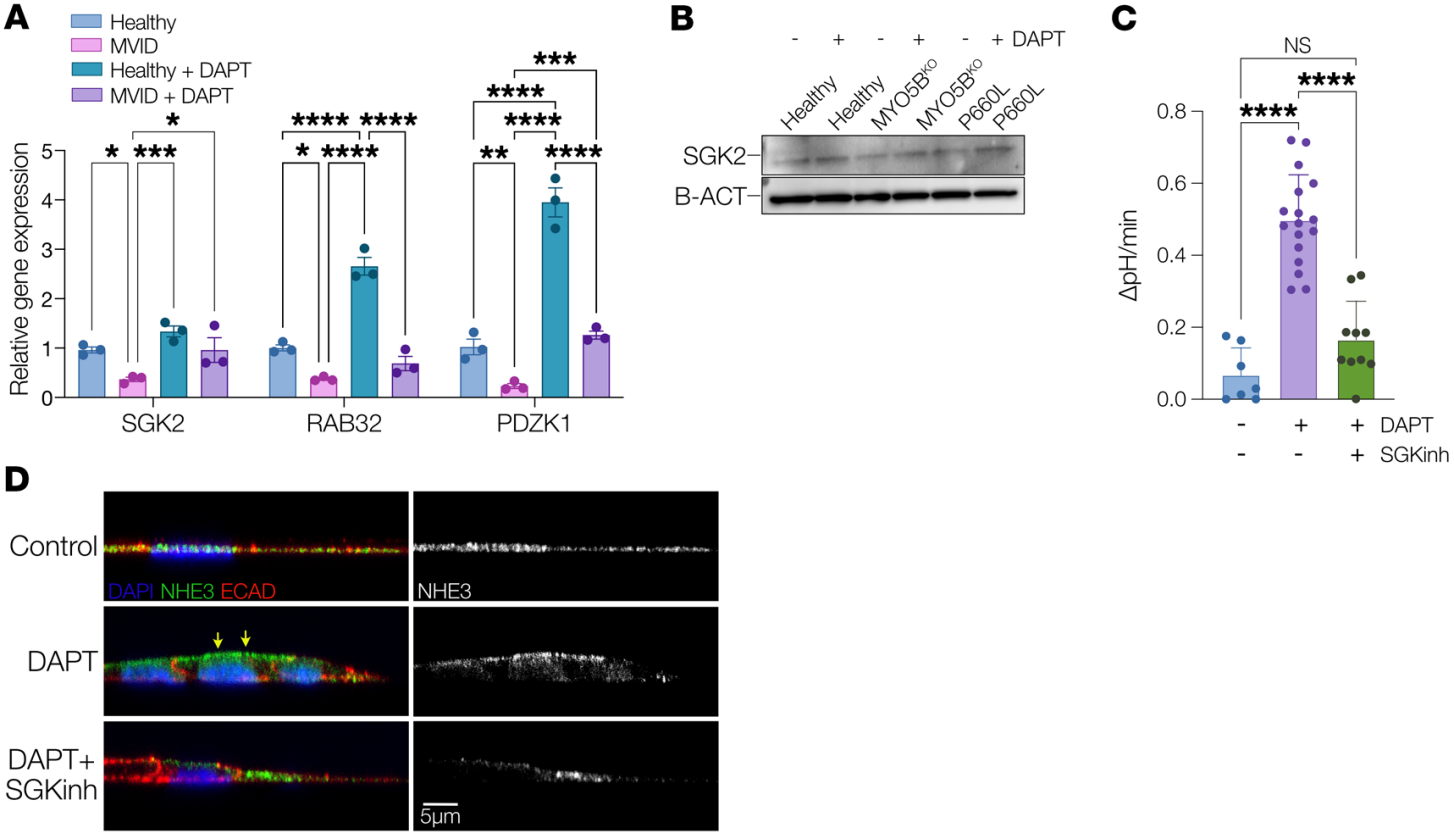
Analysis of genes potentially involved in DAPT-mediated rescue of MVID enteroids. (**A**) qPCR analysis showing relative gene expression (normalized to healthy untreated) for serum/glucocorticoid-regulated kinase 2 (SGK2), Rab GTPase 32 (RAB32), and PDZ domain–containing 1 (PDZK1) in healthy and MVID enteroids with and without DAPT (10 μM). Data are shown as means ± SEM; *n* = 3 experiments. (**B**) Immunoblot of SGK2 protein changes with and without DAPT. (**C**) Summary graph showing NHE3-dependent pH changes in MVID patient enteroids with and without DAPT (10 μM) and MVID patient enteroids with and without DAPT and SGK inhibitor (GSK650394, 5 μM). Data are shown as means ± SEM; *n* = 3 experiments; ****P < 0.0001; 2-way ANOVA with Tukey’s post hoc testing. (**D**) *Z*-plane confocal images in P660L patient enteroids with and without DAPT (10 μM) and MVID patient enteroids with and without DAPT and SGK inhibitor (GSK650394, 5 μM) showing NHE3 (green), E-cadherin (red), and nuclei (blue) with composite (left) and NHE3 only (grayscale; right).

Given the evidence for functional and structural recovery in MVID cells with different mutations following γ-secretase inhibition, we sought to identify potential pathways and target proteins that may mediate the effect. We therefore conducted unbiased bulk RNA sequencing of healthy versus MVID enteroids (MYO5B KO) with and without DAPT treatment. Figure 7, A–C, shows the large number of up- and downregulated genes associated with DAPT treatment as well as MYO5B loss of function, with both MVID and healthy transcriptomes showing similar global changes following DAPT treatment (Supplemental Figure 4). To detect genes of interest, we identified genes (Figure 7D, dashed boxes) that were significantly altered at baseline between MVID and healthy enteroids and that were significantly altered in the opposite direction following DAPT treatment (e.g., downregulated in MVID at baseline and upregulated following DAPT treatment). To further prioritize, we filtered these hits based on log_2_ fold change, false discovery rate (FDR), and base mean (Figure 7E). Gene Ontology and protein interaction analysis (Figure 7F and Supplemental Figure 5) implicated genes involved in transport regulatory activity, metabolic processes, and, interestingly, PPARα signaling. The top upregulated gene hits in our prioritized analysis included *RAB32*, *SGK2*, *PDZK1*, *ANPEP*, and *ANXA13*. ANPEP is an apical membrane aminopeptidase, and ANXA13 is an annexin family member associated with the differentiated villus enterocytes, and therefore we reasoned that these likely reflected recovery of the brush border in keeping with our structural analysis. SGK2, RAB32, and PDZK1 have been implicated in regulation of trafficking and/or alteration of membrane transport function. We therefore validated these changes by probe-specific quantitative PCR (qPCR) analysis (Figure 8A). The activity of the serum/glucocorticoid-regulated kinase (SGK) family of enzymes has been previously implicated in MVID and MYO5B function (17). Immunoblot of SGK2 protein (Figure 8B) indicated a DAPT-induced increase in SGK2 in both MVID patients’ cells consistent with the RNA expression data. To functionally assess whether SGK2 may be a potential target for recovery of MYO5B function, we tested NHE3 activity. DAPT-mediated increases in NHE3 activity in MVID cells were inhibited by pretreatment with an SGK inhibitor, suggesting that SGK function may play a mechanistic role in DAPT-mediated epithelial recovery in MVID.

## Discussion

Intestinal epithelial MYO5B is a critical regulator of epithelial polarity and the establishment of apical membrane recycling and apical trafficking (1, 3, 11, 12). Loss of MYO5B function, associated with MVID, results in a structurally abnormal brush border in patients and mouse models, with decreased localization of brush border transporters important for intestinal fluid and nutrient absorption and homeostasis. Enteroids and tissue derived from both MVID patients described here showed broadly similar phenotypic features found in previous descriptions of MVID patient tissues, with loss of microvilli and loss of transport protein localization (16, 18). Our MxIF method allowed further analysis of protein localization and identification of differences in localization patterns in tissue between the 2 patients reflective of the impact of different *MYO5B* mutations. Previous studies have shown that the P660L missense mutation induces a rigor status in MYO5B (10). The second MVID tissue and enteroids reported here possess 2 severe truncating mutations, which likely lead to deletion of MYO5B.

In addition to structural and localization changes, we directly investigated transport function in MVID and healthy epithelial cells. There are minimal data on electrolyte transport function in patient-derived human enteroids, and existing data have been restricted to adult samples (30). We found reduced SGLT1-dependent Na^+^ transport and near-complete loss of NHE3 transport with normal chloride secretion in MVID cells. Previous studies in MYO5B-KO Caco-2 cells, a pig model of MVID (P660L), and *Myo5b*-KO mice have suggested maintenance of chloride and therefore fluid secretion in MVID (15, 17, 18). Our data corroborate these findings and suggest that loss of Na^+^-mediated transport, particularly via NHE3, may underlie the majority of the fluid loss. Quantitatively, most of the fluid (5 L/d in an adult) required to be absorbed (primarily by Na^+^-dependent mechanisms) in the small intestine is generated endogenously by the gastrointestinal tract. Strategies to enhance or prevent loss of NHE3 function may therefore have considerable benefit for therapy in MVID. An interesting finding was the increased proportion of putatively calcium-activated chloride channel versus CFTR activity in young children, likely reflecting developmental differences in duodenal transport. Further studies to carefully characterize the effects on transport of factors such as developmental age, sex, and environment, in healthy cells, are needed to provide normative data to understand changes seen in disease states such as celiac disease, inflammatory bowel disease, and environmental enteric dysfunction.

The maintenance of normal chloride and fluid secretion in the setting of loss of Na^+^ transport likely further exacerbates fluid loss at baseline, but particularly during periods of stress such as infection where chloride channel secretion is stimulated. To assess a potential immediate therapeutic intervention, we conducted preclinical studies using the approved antidiarrheal crofelemer, a combined CFTR and calcium-activated chloride channel blocker. Short-circuit current analysis showed robust inhibition of stimulated chloride currents with a higher maximal inhibition than previously reported. Previous data in T84 cells suggest that crofelemer has increased potency on the putative calcium-activated chloride channel versus CFTR (31), and the age-dependent differences we found in these currents may underlie this finding. If so, crofelemer would be predicted to be more effective in young children than adults. Crofelemer was also able to inhibit maximally stimulated fluid secretion significantly, but incompletely, in enteroids. Fluid management of MVID patients is a prominent clinical issue, and even a modest (10%–20%) decrease in intestinal fluid output may translate to meaningful symptomatic improvement. Further clinical trials are needed to assess whether this may translate to clinical benefit for MVID patients.

We found that MVID enteroids exhibited altered differentiation characteristics versus healthy controls consistent with previous mouse *Myo5b*-KO data showing disrupted stem cell marker expression, a loss of tuft cells, and MYO5B loss–associated decreased transcription levels of Wnt ligands with maintained expression of Notch signaling molecules (24). The balance of proliferation and differentiation in the intestinal epithelium is a complex process that is driven by the balance of stem cell activity and relative specification to the various mature cell types (secretory cell vs. enterocyte) and governed in tissue by the balance of niche growth factor signals (23). In this regard, enteroid models are an inherently regenerative model, where the exogenous supplementation of growth factors can only approximate the endogenous tissue state. Therefore, functional and transcriptomic enteroid data should be interpreted in the setting of the specific growth conditions present in a particular experiment. However, an advantage of the system is the ability to directly manipulate growth factors to direct epithelial cell state and compare changes. Given the previous data suggesting imbalanced Wnt/Notch signaling with MYO5B loss and initial evidence of enhanced epithelial differentiation induced by a γ-secretase inhibitor, dibenzazepine, in a *Myo5b*-KO mouse model (24), we used γ-secretase inhibition to test the possibility of functional recovery in patient enterocytes. γ-Secretase inhibition via DAPT induced significant increases toward differentiation in all cell lineages in healthy enteroids and partially recovered the defective differentiation seen in MVID enteroids grown in standard differentiation medium. Notably, DAPT-treated MVID epithelial cells showed structural evidence of increased polarization and maturation with increases in cell height, microvillus height, and brush border expression of NHE3. γ-Secretase, while well known for a key role in Notch signaling, has a large and diverse set of cellular targets impacting a wide range of signaling and other cellular processes (32). Given the complexity and variety of the γ-secretase substrate repertoire, it is likely that the rescue of structure and function demonstrated in MVID cells is not solely due to effects on Notch signaling. Importantly, however, we have demonstrated in MVID patient–derived enteroids that the epithelial defects in MVID can be reversed and that loss of MYO5B function can be bypassed by targeting of pathways affected by γ-secretase inhibition.

To start to understand the basis of DAPT-mediated rescue of epithelial function in MVID, we carried out transcriptomic analysis of DAPT-treated enteroids. DAPT treatment alone on healthy enteroids caused a large shift in the transcriptomic landscape with over 8,000 genes significantly changed. We narrowed down potential candidate pathways by focusing on genes altered at baseline in MVID versus healthy enteroids, and subsequently changed in the opposite direction by addition of DAPT. Using this strategy, we identified several genes that may potentially be involved in the functional recovery, and further interrogation of this data set will likely provide insights for both γ-secretase–dependent and MYO5B-dependent intestinal epithelial processes. We focused on several genes that may be plausibly involved in cellular pathways related to MYO5B, based on previous data and known functional roles.

An interesting identified target was serum/glucocorticoid-regulated kinase 2 (SGK2), which was downregulated in MVID patient enteroids relative to healthy controls, but significantly upregulated upon DAPT treatment. SGK2 is part of a family of closely homologous kinases (SGK1–SGK3) involved in cell stress responses (33, 34). SGK function in epithelial cells is thought to be involved in regulation of apical electrolyte transport, with evidence of regulation of sodium reabsorption in the kidney and direct regulation of NHE3 activity via NHE3 regulatory factor (NHERF) proteins (35, 36). SGK expression is regulated by many transcription factors, including AP-1, NF-κB, GATA, Ets-2, and p53, and there is one report relating to Notch signaling in macrophages (37–40). In kidney, SGK2 but not SGK1 regulates NHE3 expression and activity by altering the stability of the transporter at the apical membrane (36, 41). In terms of linking SGK function and MYO5B, there is evidence that regulation of SGK1 activity can be mediated by interaction with the NHERF complex via phosphoinositide-dependent protein kinase 1 (PDPK1), linking SGK function with activity and localization of apical transport (42). PDPK1 has been directly linked with MYO5B function with studies suggesting that PDPK1-dependent signaling may provide a therapeutic target for treating MVID (17). Given the suggestive evidence in the literature, our findings that both expression and protein abundance of SGK2 are upregulated by DAPT, and that inhibition of SGK activity can block rescue of NHE3 function, provide some preliminary mechanistic data of SGK2 involvement in bypassing the effects of MYO5B loss of function.

Other candidates potentially involved in regulation of apical trafficking and protein localization and identified in our transcriptomic screen include Rab GTPase 32 (Rab32) and PDZ domain–containing 1 (PDZK1). Rab32 is a multifaceted regulator linked to multiple cellular functions, including endosomal trafficking, mitochondrial dynamics, and biogenesis of lysosome-related organelles (43–45). Rab32 regulates these cellular pathways through interacting proteins that include the AP-1 and AP-3 adaptor protein complexes and may provide an alternative vesicular pathway for apical trafficking. PDZK1, also known as NHE regulatory factor 3 (NHERF3), is a PDZ domain scaffolding protein that mediates plasma membrane localization and regulation of proteins, and most notably is part of the regulatory complex for NHE3. PDZK1 is thought to form heterodimers with other NHERF family members (NHERF1 and NHERF2) and plays a role in regulation of NHE3 localization and function as well as formation and maintenance of the microvillus structure (46–48). Our studies therefore reveal several lead targets, including SGK2 and PDZK1, that can form the basis for future studies of the specific mechanism(s) involved in γ-secretase inhibition–based rescue in MVID and for future targeting of therapies.

In summary, our studies show that patient-derived enteroids recapitulate the cellular phenotype of MVID epithelial cells and provide functional and structural data in human patient cells, including identifying key ion and fluid transport changes present in MVID. We leveraged this unique resource to assess potential therapeutic approaches for symptomatic management and show that it is therapeutically feasible to bypass trafficking blockades induced by MYO5B loss of function.

## Methods

### Whole-exome and Sanger sequencing

DNA from patient 1 and mother was sent to GeneDx for whole-exome sequencing (WES) (PMID: 32655885) (9). A sequencing library was prepared using the Illumina Exome Enrichment Protocol, and captured libraries were sequenced on Illumina HiSeq 2000 or 4000. Sequences were aligned to the human genome reference sequence (hg19) using Burrows-Wheeler Aligner (BWA v0.7.15; https://sourceforge.net/projects/bio-bwa/), and variants were called with Gatk best practices (version 3.7). The data were filtered to include variants with an allele frequency of <0.001 in publicly available normative databases (Genome Aggregation Database [gnomAD]). Variants were identified using the NextCode Genuity platform. DNA from patient 2 was sent for WES via a targeted gene panel. Physiochemical differences between the canonical and patient amino acid sequences were determined using Combined Annotation Dependent Depletion (CADD; https://cadd.gs.washington.edu), and PolyPhen-2 (http://genetics.bwh.harvard.edu/pph2/), MutationTaster (https://www.mutationtaster.org), and Sorting Intolerant from Tolerant (SIFT; https://sift.bii.a-star.edu.sg) were also used to estimate the impact of the variant on DNA and protein level. The identified *MYO5B* variants were confirmed by Sanger sequencing. The relevant portion of the gene was PCR amplified, and Sanger sequencing was performed. The bidirectional sequence was assembled, aligned to reference gene sequences based on human genome build GRCh37/UCSC hg19, and analyzed for known familial sequence variant(s). Sequence alterations were reported according to the Human Genome Variation Society nomenclature guidelines.

### Materials and reagents

Forskolin and carbachol were purchased from Sigma-Aldrich. Crofelemer drug substance was provided by Napo Pharmaceuticals. Antibody against SGK2 was purchased from Cell Signaling Technology [SGK2 (D7G1) rabbit mAb, catalog 7499S]. GSK650394, the inhibitor against SGK2, was purchased from Selleck Chemicals (catalog S7209). SNARF-5F (and -6) carbolic acid, acetoxymethyl (AM) ester, acetate, was purchased from Thermo Fisher Scientific (catalog S23923), and CFTR_inh_-172 and CaCC_inh_-A01 were purchased from Tocris.

### Enteroid culture

Duodenal biopsies were obtained and cultured using methods modified from Sato et al. (25). Briefly, crypts were dissociated from duodenal biopsies obtained from patients with *MYO5B* mutation or from an age-matched healthy control patient. Isolated crypts were suspended in Growth Factor Reduced Phenol Red-Free Matrigel (Corning) and plated as 50 μL domes in a tissue culture–treated 24-well plate (Thermo Fisher Scientific) with growth factor–supplemented (Wnt, R-spondin, Noggin) medium (see Supplemental Methods for medium composition and detailed culture methods). Enteroid cultures were passaged by removal of Matrigel with Cell Recovery Solution (Corning), mechanical dissociation of enteroids, and replating in Matrigel every 4 days. For DAPT treatments, enteroids were grown in expansion medium (2–3 days) and then switched to differentiation medium with or without DAPT (10 μM). For enteroid experiments, experimental replicates are defined as separate cultures of enteroids derived from the same patient biopsy.

For electrophysiological assessments, enteroids were grown on collagen-coated 0.33 cm^2^ Transwell inserts (CLS3472, Costar Corning) and incubated in 5% CO_2_ at 37°C for at least 7 days. Medium was changed every 3–4 days. Transepithelial electrical resistance was measured using an epithelial volt/ohm meter (EVOM; World Precision Instruments).

### Electron microscopy

Enteroids were embedded in low–melting point sea plaque agarose (Cambrex Bio Science), creating faux tissue blocks. Samples were then immersion-fixed in 2.5% glutaraldehyde (Electron Microscopy Sciences), 2% formaldehyde (Electron Microscopy Sciences) in 0.1 M sodium cacodylate (Sigma-Aldrich) at pH 7.4 for at least 1 hour at room temperature and then at 4°C overnight. Samples were washed with 0.1 M sodium cacodylate, and then postfixed for 1 hour at 4°C in 1% osmium tetroxide (Electron Microscopy Sciences) in 0.1 M sodium cacodylate. Samples were then washed in deionized water and incubated in 2% aqueous uranyl acetate (Electron Microscopy Sciences) overnight at 4°C. The following day, samples were washed with deionized water and then dehydrated at 4°C in a graded ethanol series. The agar blocks were then brought to room temperature and dehydrated with 100% ethanol (Sigma-Aldrich) followed by propylene oxide (Electron Microscopy Sciences). Infiltration in LX112 resin (Ladd Research Industries) was followed by embedding in flat-bottom BEEM capsules (Electron Microscopy Sciences). The resulting blocks were sectioned using a Leica Ultracut E ultramicrotome (Leica Microsystems), and the sections were placed on grids coated with formvar (Electron Microscopy Sciences) and carbon. The sections were contrast stained with 2% uranyl acetate followed by lead citrate (Sigma-Aldrich), and imaged in a JEOL 1400 transmission electron microscope equipped with a Gatan Orius SC1000 digital CCD camera.

### Confocal, STED, and light-sheet imaging

Confocal imaging was conducted using a Zeiss 880 Airyscan microscope with Zen Black and Blue for control and analysis (Zeiss Instruments). Stimulated emission depletion (STED) imaging was conducted using an Abberior StedyCon system (Abberior Instruments GmbH) with post-image deconvolution using Huygens Pro (Scientific Volume Imaging). Light-sheet imaging was conducted using an ASI DiSPIM microscope (Applied Scientific Instruments).

### Multiplex immunofluorescence

FFPE slides were deparaffinized and incubated in Trilogy (Sigma-Aldrich) for antigen retrieval. The slides were coverslipped with 50% glycerol in 0.1 M PBS containing 1 μM Hoechst 33342, and whole slide images were scanned using a Leica/Aperio Versa 200 with ×20 objective (Leica Biosystems) for acquisition of autofluorescence signals of the tissues. Coverslips were gently removed, and the slides were preblocked with Dako serum-free protein blocking solution (X0909) for 1 hour at room temperature or overnight at 4°C. Primary conjugated antibodies listed in Supplemental Methods were applied to the slides for 1 hour at room temperature. Some antibodies were labeled with Zenon rabbit IgG labeling kits (Invitrogen) according to the manufacturer’s instruction. Fluorescence signals were quenched by carbonate buffer (pH 11) immediately after scanning of staining signals. Background signals were imaged after each quenching step and subtracted from the following staining images.

### Multiplex imaging analysis

Background-corrected immunofluorescence images were used for all multiplex image analysis.

#### Percentage of enteroendocrine and tuft cells.

This set of analyses was carried out using Fiji ImageJ. The DAPI channel was first histogram normalized, and the number of nuclei was determined by the Find Maxima function with a prominence parameter of 15. Analogously, the numbers of endocrine and tuft cells were determined using the channels for phospho-EGFR and chromogranin A, respectively, with a prominence factor of 40 and 100, respectively.

#### Measuring Feret distance.

We used a home-written Matlab code for this analysis. The CD10 was thresholded, binarized, and finally skeletonized with a minimal branch length of 10 pixels to avoid background. The maximum caliper of the Feret distance for each skeleton in the image was calculated.

#### Measuring correlation between all channels.

The correlation coefficient for each channel with every other channel was calculated using Matlab. The correlation matrix was imported into R and hierarchically clustered and plotted using the Corrplot library.

### Short-circuit current measurement

Following the formation of an enteroid monolayer, medium was removed, and the cells were rinsed and bathed in buffer solution (in mM: 130 NaCl, 0.47 KCl, 0.124 MgSO_4_, 0.33 CaCl_2_, 10 HEPES, 2.5 NaH_2_PO_4_, 10 dextrose). Custom-made chambers were designed and built to measure short-circuit current in 0.33 cm^2^ Transwell inserts (www.thiagarajahlab.com/tools/). The cells were maintained at 37°C, short-circuit current was measured using a VCCMC8 multichannel voltage clamp (Physiologic Instruments), and LabChart (ADInstruments) was used to record measurements.

### Na+/H+ exchanger measurement

Na^+^/H^+^ exchanger measurements were done using a variation of previously optimized protocols (30, 49). Briefly, enteroids were plated onto coverslips and cultured under differentiation medium conditions (Supplemental Methods). Cells were loaded with SNARF-5F-AM (5 μM) in 50 mM NH_4_Cl prepulse buffer for 15 minutes at 37°C, 5% CO_2_. Coverslips were mounted in a preheated chamber (37°C; Warner Instruments), and imaging was done using a ×20 air objective on a Zeiss 880 laser scanning microscope at 488 nm excitation, 580/640 nm emission. For each condition, fluid was aspirated and replaced with 1 mL fresh buffer maintained at 37°C. Images were collected after incubation in Na^+^-free tetramethylammonium chloride buffer and at various intervals in modified Krebs Na^+^ buffer. Intracellular pH was calibrated at the end of each experiment by exposure to 20 μM nigericin for 10 minutes in K^+^ clamp buffers to set the pH at 6, 7, and 8.

### Gene expression analysis by qPCR

Total RNA was extracted from cells using the RNeasy Mini Kit (Qiagen). Cell pellets were lysed in Buffer RLT and processed according to the manufacturer’s protocol. Total RNA concentrations were measured by absorbance at 260 nm, and quality was assessed by A260/A280 ratios. cDNA was synthesized from 1 μg of RNA, including DNA elimination step, using QuantiTect Reverse Transcription Kit (Qiagen) according to the manufacturer’s protocol.

Target transcripts were amplified using the primers listed in the Supplemental Methods (Integrated DNA Technologies) and Sso Advanced Universal SYBR Green Supermix according to the manufacturer’s protocol (Bio-Rad). All qPCR reactions were assayed in triplicate for each sample, and the average quantification cycle (Cq) value was used to calculate the mean expression ratio of the test sample compared with the control sample using the 2^–ΔΔCt^ method. Cq values for targets were analyzed relative to Cq values for the GAPDH housekeeping gene. The sequences for the qPCR primers were designed using Primerbank (Supplemental Methods) and purchased from Integrated DNA Technologies.

### Bulk RNA sequencing analysis

The paired RNA sequencing reads were preprocessed using fastp. The filtered reads were then mapped to the GRCh41 genome assembly using HISAT2 with an overall alignment greater than 90%. The mapped transcripts were quantified using Salmon, and differential analysis was performed using DESeq2 with a significance FDR cutoff of 0.05. All subsequent analysis and filtering were carried out using custom-written programs in Matlab (50–53).

### Statistics

Significance was assessed using 2-tailed *t* test or 2-way ANOVA with post hoc multiple-comparison testing (Tukey’s), and, where indicated, *P* less than 0.05 was considered significant. Graphs were generated using GraphPad Prism 8.

### Study approval

The Institutional Review Boards of Boston Children’s Hospital and Phoenix Children’s Hospital approved this study (BCH IRB P00027983, PC IRB 10-019), and informed consent/assent was given in accordance with the Declaration of Helsinki.

### Data availability

RNA sequencing data can be accessed via the NCBI’s Gene Expression Omnibus database (GEO GSE240928; https://www.ncbi.nlm.nih.gov/geo/query/acc.cgi?acc=GSE240928). Multiplex immunofluorescence data can be viewed via the Pediatric Congenital Diarrhea and Enteropathy Consortium (PediCODE) Atlas (https://pedicode.org/database-viewer/code-atlas-database/). A Supporting Data Values file with all reported data values is available as part of the supplemental material.

## Author contributions

The studies and manuscript were conceptualized by JRT, JRG, and IK. Development of methodology was performed by JRT, KR, HO, JTR, MTB, and SH. Clinical investigation and data collection were carried out by LJ, MDS, and JDG. Experiments were performed by MK, KR, HO, MTB, EK, IK, and JRT. Manuscript writing and editing was supervised by JRT and reviewed by all authors.

## Supplementary Material

Supplemental data

Supporting data values

## Version 1 08/29/2023

In-Press Preview

## Version 2 10/16/2023

Electronic publication
