# Supplementary material for: Patient-derived enteroids provide a platform for the development of therapeutic approaches in microvillus inclusion disease
Source: J Clin Invest. 2023 Oct 16;133(20):e169234. doi: 10.1172/JCI169234 (PMC10575727; doi:10.1172/JCI169234)
Supplement: Supplemental data [file jci-133-169234-s192.pdf]

Supplementary Materials and Methods

Patient Clinical Summaries

Patient 1: Male with microvillus inclusion disease who is sustained on parenteral nutrition (PN). Patient was diagnosed within 2 months of age secondary to intractable diarrhea with need for PN support. Endoscopic evaluation was notable for villus blunting and abnormal CD10 brush border staining. Patient's medical course has been complicated by oral aversion, central line associated bloodstream infection, iron deficiency anemia, electrolyte derangements, and PN associated liver disease. Endoscopic biopsies for enteroid generation obtained at age 3 yrs. His PN support requires sodium supplementation (ranging from 14-17 mEq/kg/day) as well as maximum acetate provision and potassium infusion (0.25 mEq/kg/h rate due to high gastrointestinal losses.

Patient 2: Female born at 39 weeks gestation with onset of severe secretory diarrhea within the 1st week of life associated with acidosis and severe dehydration. EGD performed at 1 month consistent with MVID (gross blunting of villi, microvillous inclusions on EM). Patient remained PN dependent until bowel transplant (age 5) with biopsies retained for enteroid generation. Underwent multivisceral transplant (liver, pancreas, small intestine and colon) with retained native duodenum. Patient alive 4 years post-transplant.

Enteroid Media and Culture

Expansion Media:

Component	Volume	Catalog #	Final Concentration
L-WRN Conditioned Media	65 mL	ATCC CRL-3276	65%
Advanced DMEM/F12	30 mL	Gibco 12634-028	30%
GlutaMax (100X)	1 mL	Gibco 35050-061	1%
HEPES 1M	1 mL	Gibco 15630-080	10 mM
Primocin	200 µL	Invivogen ant-pm-2	0.2%
Normocin	200 µL	Invivogen ant-nr-2	0.2%
B27	1 mL	Gibco 12587010	1%
N2	500 µL	Gibco 17502-048	0.5%
Nicotinamide 1M	1 mL	Sigma N0636	10 mM
N-Acetyl-Cysteine (500 mM)	100 µL	Sigma A8199	500 µM
A 83-01 (500 µM)	100 µL	Sigma SML0788	500 nM
SB202190 (5 mg/505 µL)	33.2 µL	Sigma S7067	

EGF (500 µg/mL)	10 µL	Peprotech 315-09	50 ng/mL
Gastrin (500 µM)	10 µL	Sigma G9145	10 nM
Prostaglandin E2 (5 mg/mL)	1 µL	Sigma P5640	100 nM
Y-27632 (3.2 mg/mL)	100 µL	Sigma Y0503	10 µM
**Total Volume**	**100 mL**		

Differentiation Media:

**Component**	**Volume**	**Catalog #**	**Final Concentration**
L-WRN Conditioned Media	15 mL	ATCC CRL-3276	15%
Advanced DMEM/F12	80 mL	Gibco 12634-028	80%
GlutaMax (100X)	1 mL	Gibco 35050-061	1%
HEPES 1M	1 mL	Gibco 15630-080	10 mM
Primocin	200 µL	Invivogen ant-pm-2	0.2%
Normocin	200 µL	Invivogen ant-nr-2	0.2%
B27	1 mL	Gibco 12587010	1%
N2	500 µL	Gibco 17502-048	0.5%
Nicotinamide 1M	1 mL	Sigma N0636	10 mM
N-Acetyl-Cystein (500 mM)	100 µL	Sigma A8199	500 µM
EGF (500 µg/mL)	10 µL	Peprotech 315-09	50 ng/mL
**Total Volume**	**100mL**		

Plating on Transwells:

Formed enteroids were removed from tissue culture plates and Matrigel® was dissolved in Cell-recovery solution (Corning). Enteroids were dissociated by vigorous pipetting and incubation at 37ºC with TRIPL-E (ThermoFisher) for 2-3 mins. Cells were plated onto human placental collagen IV [Please confirm collagen type] (Sigma)-coated Transwell filters (Corning) with 0.3-µm-pore size inserts and cultured for 2-4 days in Expansion media including Rho Kinase inhbitor (Y-27632) until transepithelial resistance (TEER) started to rise. Media was switched to the differentiation media and electrophysiological and immunohistological measurements done after 10-12 days, when TEER reached to >2000Ω/cm^2.

Plating on Coverslips:

Formed enteroids were removed from tissue culture plates and Matrigel® was removed in Cell-recovery solution. Enteroids were dissociated by vigorous pipetting and incubation at 37ºC with TRIPL-E for 2-3 mins. Cells were plated onto human placental collagen coated coverslips and cultured in the differentiation media ± DAPT for four days.

Enteroid Formation Assay

Formed enteroids (P1-3) were removed from tissue culture plates and Matrigel® was removed in cell-recovery solution. Enteroids were dissociated by vigorous pipetting and incubation at 37ºC with TRIPL-E for 2-3mins. Cells were counted and re-plated in Matrigel® for MVID and healthy enteroids at approximately same density. Cells were cultured in the expansion media for 3 days and formed enteroid numbers were counted in each plate well.

Enteroid Swelling Assay

Enteroid swelling after Crofelemer (100 µM) or vehicle treatment (PBS) was performed as previously described (1). In brief, Crofelemer or vehicle (PBS) was administered 30mins prior to forskolin 10µM. Enteroids were imaged every 10mins for 2hours using anautomated plate imaging system (Biotek Cytation 5, Agilent Santa Clara, CA). Measurements of cell diameter were carried out using Image J with the Object J plugin.

EM image analysis

EM images (at least 10-15 per enteroid) were analyzed blinded in Image J for measurement of microvilli and actin bundle length and distance between apical membrane and the majority of cell organelles.

qPCR primer sequences

PCR primer sequences:

Human SGK2;
Primer 1: 5'- CCACGGACTTCGACTTCCTC -3'

Primer 2: 5'- GTGCCGCACGTTCTTCAGA -3'

Human PDZK1:
PrimeTime™ Predesigned qPCR Assays (Assay Id: Hs.PT.58.4953162)

Human RAB32:
Primer 1: 5'- CAGGTGGACCAATTCTGCAAA -3'

Primer 2: 5'- GGCAGCTTCCTCTATGTTTATGT -3'

MxIF Antibodies

ACTG1	sc-65638 AF488	AB_2890619	1:100
Beta-catenin	NBP1-54467IR	12F7	1:50
CD10	sc-46656 AF488	AB_2890648	1:100
CHGA	NBP2-47850IR	CGA/493	1:2000
Defensin 5A	NB110-60002IR	8c8	1:200
Cd26/DPP4	NBP2-70588C	OTI11D7	1:200
EGFR pY1068	ab205828	AB_2890267	1:200
Ep-CAM	ab275122	EPR677(2)	1:100
GLUT2	NBP2-22218AF647	AB_2890913	1:50
LAMP2A	ab282009	EPR4207(2)	1:50
MYO5B	NBP1-87746	AB_11034537	5 μg/ml (Zenon labeled)
pNHE3	NB110-81529R	14D5	1:50
SGLT1	NBP2-38748	AB_2890609	5 μg/ml (Zenon labeled)
Villin	sc-58897 AF488	1D2C3	1:50

Supplemental Acknowledgments

PediCODE Consortium Members:

Martín G. Martín, University of California, Los Angeles
Wayne Lencer, Boston Children's Hospital
Aleixo Muise, The Hospital for Sick Children, Toronto
Yaron Avitzur, The Hospital for Sick Children, Toronto
Sari Acra, Vanderbilt University Medical Center

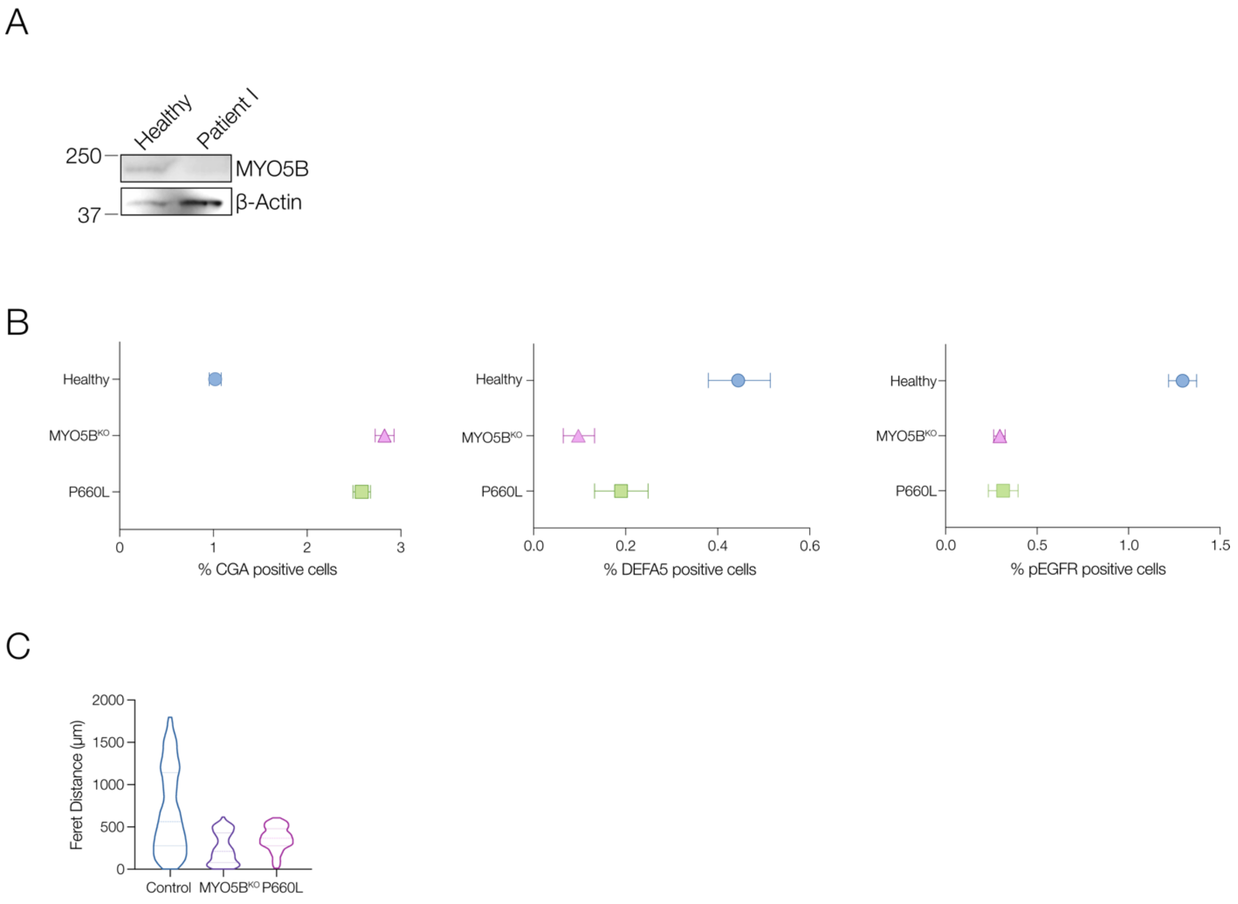

Supplementary Figure 1

SUPPLEMENTARY FIGURE 1: A. Immunoblot of MYO5B in healthy control sample and Patient 1. **B**. Graphs showing whole biopsy counts of positive cells for Chromogranin A (CGA), Defensin alpha 5 (DEFA5) and phospho-Epidermal Growth Factor Receptor (pEGFR). Error bars represent means ± SD, n=3-4 sections **C.** Graph of continuity analysis (Feret's Distance) of linear CD10 staining across all biopsy images in Fig 2.

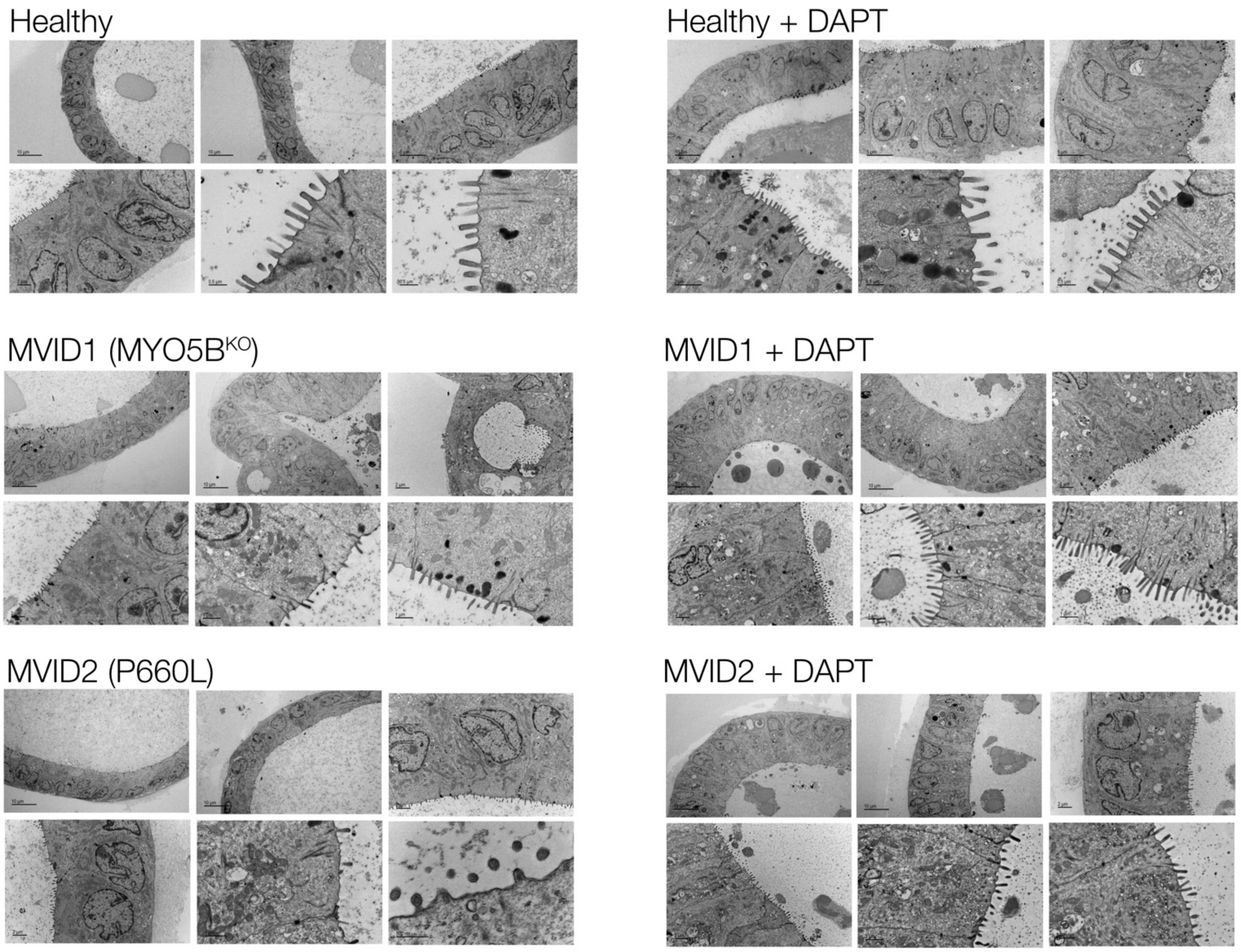

SUPPLEMENTARY FIGURE 2: Electron micrographs of healthy and MVID enteroids ± DAPT (10 μM).

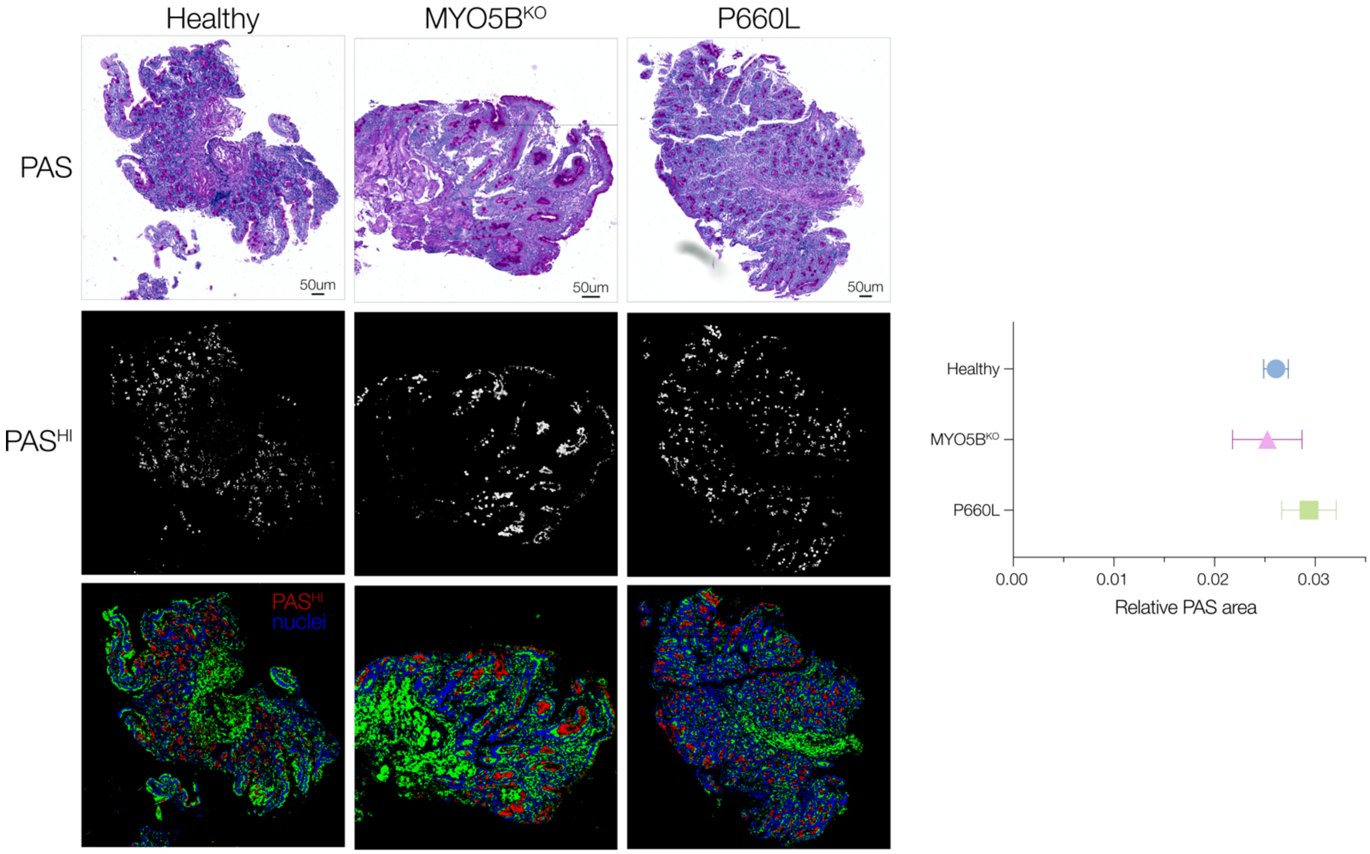

Supplementary Figure 3

SUPPLEMENTARY FIGURE 3: Immunofluorescence images of human duodenal biopsy sections stained for PAS (left) to show goblet cells, with analysis to show highly stained PAS cells (PAS^{HI}). Graph showing area of PAS^{HI} relative to total cells. Error bars represent means ± SD, n=3 sections

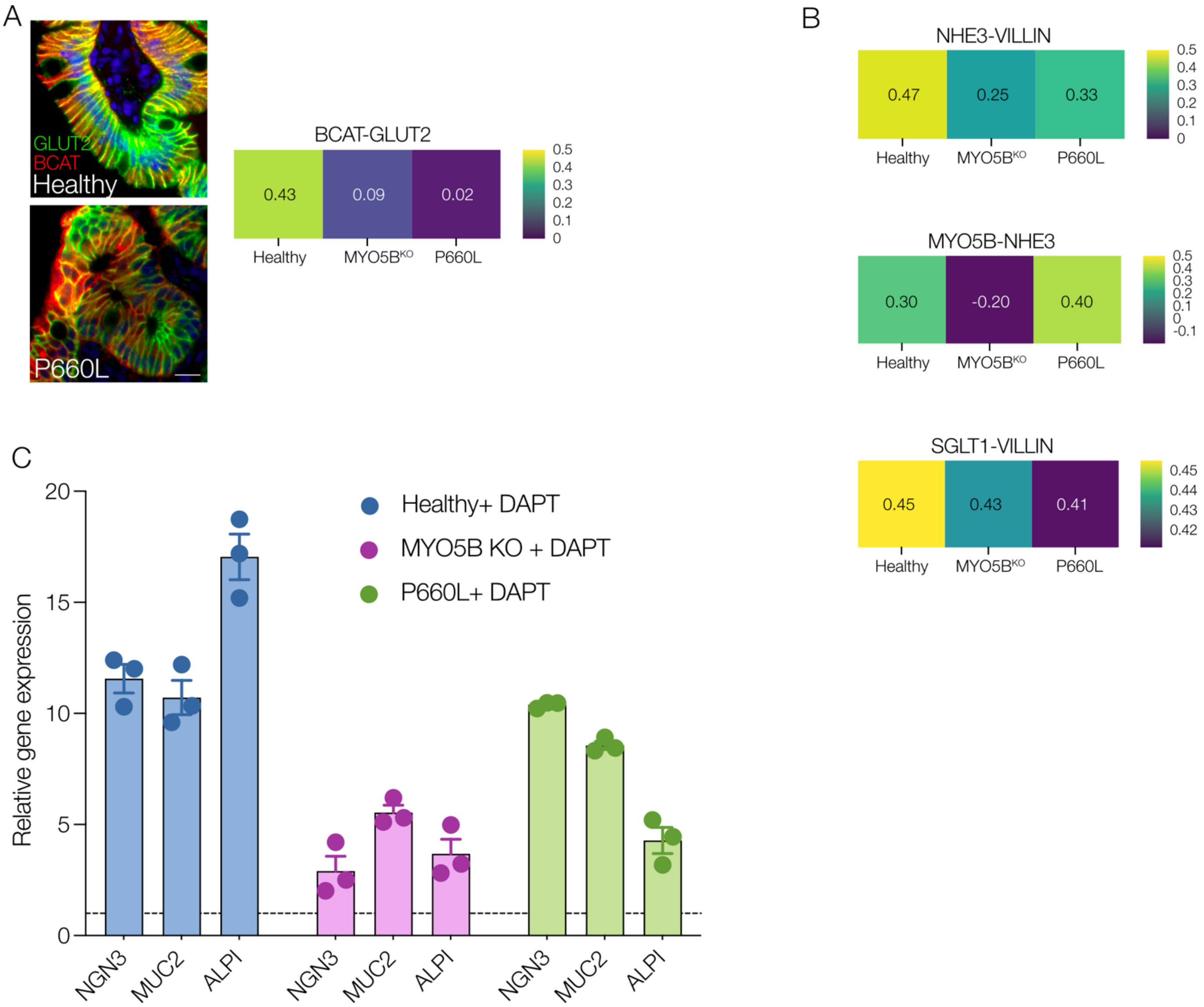

SUPPLEMENTARY FIGURE 4: **A.** Individual pair-wise staining for Glucose Transporter 2 (GLUT2) and beta-catenin (BCAT) with heatmap of pairwise Pearson's correlation coefficient with numerical coefficient in box center. B. Heatmap of pairwise Pearson's correlation coefficient for Na^{+}/H^{+} exchanger 3 (NHE3) and Villin, Sodium-Glucose Cotransporter 1 (SGLT1) and Villin, and Myosin 5b (MYO5B) and NHE3 **C.** Relative gene expression (normalized to differentiated) for neurogenin3 (NGN), mucin 2 (MUC2) and alkaline phosphatase (ALPI) in healthy and MVID enteroids following addition of DAPT. Dotted line indicates baseline without DAPT. Error bars represent means ± SEM, n=3 experiments.

A

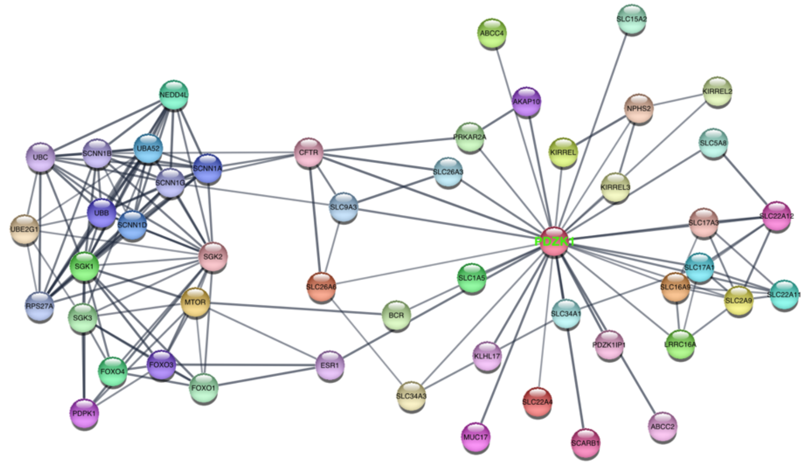

B

		"-log 10 (P-value)"
**GO:BP**		
	response to organophosphorus	2.52
	response to purine-containing compound	2.22
	positive regulation of transport	1.99
	response to fatty acid	1.79
	response to xenobiotic stimulus	1.71
	positive regulation of transporter activity	1.64
	small molecule metabolic process	1.54
	lipid metabolic process	1.44
	response to lipid	1.41
**KEGG**		
	homocysteine metabolic process	1.31
**REAC**		
	PPAR signaling pathway	3.50
	Metabolic pathways	2.14
	PPARA activates gene expression	1.85
	Regulation of lipid metabolism by PPARalpha	1.83
	Signaling by Retinoic Acid	1.45
**HPA**		
	duodenum; enterocytes - Microvilli	2.00
	duodenum; endocrine cells	1.73
	duodenum; enterocytes - Microvilli	1.55
	small intestine; enterocytes	1.49
	colon; enterocytes - Microvilli	1.31

C

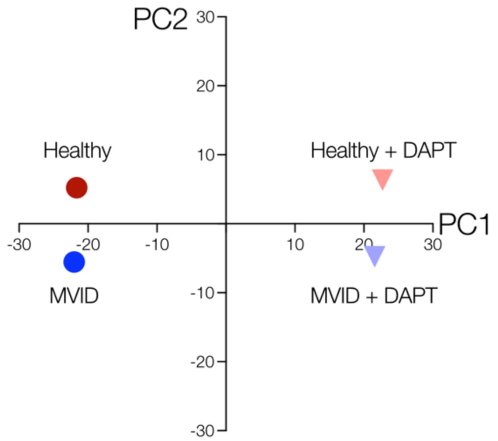

SUPPLEMENTARY FIGURE 5: A. STRING protein interaction network analysis for SGK2 and PDZK1. **B.** Pathway analysis showing most significant GO terms, HPA terms and KEGG pathways with P-values. **C.** Principal component analysis (PCA) plot showing global transcriptome changes in healthy and MVID enteroids following DAPT treatment.

SUPPLEMENTARY MOVIE 1: Lightsheet microscopy scan showing three-dimensional MVID enteroid stained for actin (white) and nuclei (blue) indicating abnormal intracellular large inclusions with microvilli.
